# A bHLH121/URI–ANAC017–MAT1/SAM1 regulatory cascade controls iron homeostasis in *Arabidopsis*

**DOI:** 10.1111/tpj.71157

**Published:** 2026-10-14

**Authors:** Chunyan Tu, Qi Wu, Fangwei Yu, Renfang Shen, Xiaofang Zhu

**Affiliations:** ^1^ State Key Laboratory of Soil and Sustainable Agriculture, Institute of Soil Science Chinese Academy of Sciences Nanjing 210008 China; ^2^ College of Land Resources and Environment Jiangxi Agricultural University Nanchang Jiangxi 330045 China; ^3^ Tobacco Research Institute Chinese Academy of Agricultural Sciences Qingdao 266101 China; ^4^ Institute of Vegetable Crops Jiangsu Academy of Agricultural Sciences Nanjing 210014 China

**Keywords:** *Arabidopsis*, NAC domain containing protein 17, methionine adenosyltransferase, cell wall, Fe uptake/transport, Fe reutilization

## Abstract

Iron (Fe) deficiency is a critical environmental constraint limiting plant growth, yield, and quality. Although plants have evolved sophisticated mechanisms to enhance Fe uptake, transport, and utilization, key components of the underlying regulatory network remain unclear. Here, we identify the NAM, ATAF1/2, and CUP‐SHAPED COTYLEDON 2 (NAC) transcription factor ANAC017 as an essential regulator of Fe homeostasis in *Arabidopsis*. *ANAC017* expression is strongly induced under Fe deficiency, and loss of *ANAC017* function leads to reduced Fe accumulation in both roots and shoots, impaired root elongation, and pronounced leaf chlorosis. Notably, *anac017* mutants show excessive Fe retention in the cell wall—particularly within the hemicellulose fraction—under Fe‐deficient conditions, suggesting that ANAC017 contributes to the mobilization and reutilization of apoplastic Fe. Mechanistically, ANAC017 functions within the canonical Fe signaling cascade. We show that the core Fe regulator, the BASIC HELIX‐LOOP‐HELIX (bHLH) transcription factor bHLH121/URI, directly activates *ANAC017* transcription, and overexpression of *ANAC017* partially rescues the Fe‐sensitive phenotype of the *bhlh121/uri* mutant. Further experiments revealed that the transcription factor ANAC017 binds to the promoter of *MAT1*/*SAM1* (encoding *S*‐adenosylmethionine synthetase), potentially modulating its expression. In conclusion, this study reveals that ANAC017 functions downstream of the bHLH121/URI pathway to regulate Fe homeostasis via two separate mechanisms: facilitating cell wall Fe reutilization and mediating *S*‐adenosylmethionine synthetase metabolism by regulating *MAT1/SAM1*.

## Significance Statement

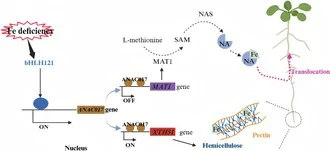

This study identifies ANAC017 as a previously unrecognized downstream effector of bHLH121/URI and demonstrates its essential role in *Arabidopsis* Fe homeostasis. ANAC017 promotes cell wall Fe remobilization and directly binds the promoter of *MAT1*/*SAM1*, which encodes SAM synthetase. Given that SAM serves as a precursor for ethylene and nicotianamine, ANAC017 may potentially influence SAM metabolism to modulate Fe homeostasis. These findings substantially extend the bHLH121/URI‐centered network and offer new insights into Fe nutrition under fluctuating environments.

## INTRODUCTION

Iron (Fe) is an essential micronutrient required for numerous physiological and metabolic processes, including photosynthesis, hormone biosynthesis and nitrogen fixation (Liang, 2022; Zhang, 2014). Fe deficiency typically manifests as interveinal chlorosis in young leaves, reduced photosynthetic capacity, and impaired growth, ultimately lowering crop yield and quality (Briat et al., 2015). Although Fe is abundant in most soils, its bioavailability is severely restricted by pH‐dependent solubility, as it predominantly exists as insoluble Fe(III) compounds (Briat et al., 2015; Zhang et al., 2021). Consequently, plant‐available Fe is often far below physiological demand, particularly in calcareous soils where Fe deficiency is most prevalent (Kobayashi & Nishizawa, 2012; Lindsay & Schwab, 1982). As neutral to alkaline soils constitute a substantial proportion of global agricultural land, Fe deficiency represents a widespread and persistent constraint on crop production. Elucidating the genetic mechanisms underlying plant adaptation to Fe limitation is therefore an essential step toward improving Fe acquisition and utilization efficiency in crops.

To cope with the limited bioavailability of Fe in soils, plants have evolved two distinct acquisition strategies: Strategy I and Strategy II (Kobayashi et al., 2018; Kobayashi & Nishizawa, 2012; Schmidt, 2003). Strategy II, characteristic of graminaceous plants, relies on the biosynthesis and secretion of mugineic acid family phytosiderophores under Fe deficiency (Bashir et al., 2010; Schmidt, 2003). These compounds form soluble Fe^3+^‐phytosiderophore complexes (Jurkevitch et al., 1993; Nozoye et al., 2015), which are subsequently transported through specific Yellow Stripe/Yellow Stripe‐like (YS/YSL) transporters (Curie et al., 2001; Lee et al., 2009). In contrast, Strategy I, employed by dicots and non‐graminaceous monocots, relies on rhizosphere acidification, reduction of ferric Fe, and uptake of ferrous Fe (Siwinska et al., 2018). In *Arabidopsis*, Fe deficiency activates plasma membrane H^+^‐ATPase to acidify the rhizosphere and enhance Fe^3+^ solubility. The ferric chelate reductase (FCR), encoded by *Ferric Reduction Oxidase 2* (*FRO2*; Robinson et al., 1999), then reduces Fe^3+^ to Fe^2+^, which is imported by Fe‐regulated transporters such as IRON REGULATED TRANSPORTER 1/2 (IRT1/2; Eide et al., 1996) in root epidermal cells (Grillet & Schmidt, 2019; Liang, 2022). Following epidermal uptake, Fe is transported upward through the xylem as Fe^3+^‐citrate chelates, a process requiring FRD3 (ferric reductase defective3; Durrett et al., 2007; Green & Rogers, 2004) to load citrate into the xylem. After being loaded into the phloem from the xylem via OPT3 (Zhai et al., 2014), Fe is further translocated and distributed as Fe^2+^‐nicotianamine (NA) complexes (Curie et al., 2009; Kumar et al., 2017) via Yellow Stripe‐Like (YSL) family transporters (Didonato Jr et al., 2004; Jean et al., 2005) and NA efflux transporters (NAET1/2; Chao et al., 2021). During this process, NA serves as a key chemical carrier for phloem Fe transport, and its deficiency renders plants unable to utilize Fe deposited in the phloem, leading to severe deficiency symptoms. NA is synthesized from *S*‐adenosylmethionine (SAM) by NAS enzymes, whereas SAM is produced from L‐methionine by *S*‐adenosylmethionine synthetase (Sekula et al., 2020), with S‐ADENOSYLMETHIONINE SYNTHETASE‐1 (*MAT1*/*SAM1*) serving as a major encoding gene. Notably, SAM is also a precursor for ethylene biosynthesis (Vanderstraeten & Straeten, 2017), and ethylene signaling is known to play critical roles in regulating Fe homeostasis (Lingam et al., 2011).

In addition to acquiring Fe from rhizosphere, plants rely on internal Fe allocation and remobilization to alleviate Fe‐deficiency‐induced leaf chlorosis (Shi et al., 2012; Zhang et al., 1991). The apoplast serves as a major Fe reservoir, storing up to 75% of total Fe in roots and about 30% in leaves under Fe‐sufficient conditions, thereby providing considerable Fe storage potential (Bienfait, 1985; Liu et al., 2023). Within this reservoir, hemicellulose and pectin are the predominant Fe‐binding components of the cell wall (Liu et al., 2023). Under Fe‐deficient conditions, plants activate and mobilize this apoplastic Fe pool through multiple regulatory mechanisms to enhance Fe‐deficiency tolerance. Increasing evidence indicates that the reutilization of the apoplastic Fe reservoir in roots is tightly linked to cell wall biosynthesis and modifications (Clemens, 2021; Peng et al., 2021; Shi et al., 2018; Ye et al., 2015). For example, the NAM, ATAF1/2, and CUP‐SHAPED COTYLEDON 2 (NAC) transcription factor ANAC050 has been shown to regulate the expression of *XYLOGLUCAN ENDOTRANSGLUCOSYLASE/ HYDROLASE 31* (*XTH31*), thereby promoting hemicellulose remodeling and facilitating the mobilization of cell wall‐bound Fe in *Arabidopsis* (Huang et al., 2025).

In plants, Fe uptake and homeostasis are tightly coordinated by an intricate transcriptional regulatory network, in which the BASIC HELIX‐LOOP‐HELIX (bHLH) family plays a central role (Gao et al., 2019). As an upstream activator of Fe signaling, bHLH121/URI is phosphorylated under Fe‐deficient conditions and forms heterodimers with bHLH34/104/105/115, thereby inducing downstream regulators such as bHLH38/39/100/101, FER‐LIKE IRON DEFICIENCY INDUCED TRANSCRIPTION FACTOR (FIT, bHLH029), POPEYE (PYE, bHLH047), BRUTUS (BTS), and several MYB‐type transcription factors including MYB72 and MYB10 (Gao, Robe, Bettembourg, et al., 2020; Gao, Robe, & Dubos, 2020; Kim et al., 2019; Lei et al., 2020; Li et al., 2016). In *Arabidopsis*, two major branches of Fe‐deficiency response are governed by FIT and PYE (Ivanov et al., 2012; Tissot et al., 2019), with FIT acting as the master regulator of root Fe uptake (Colangelo & Guerinot, 2004). It forms heterodimers with bHLH38/39/101 to activate the expression of *FRO2* and *IRT1*, thereby enhancing Fe uptake in roots (Kim et al., 2019; Kurt & Filiz, 2018; Long et al., 2010; Schwarz & Bauer, 2020). Meanwhile, PYE regulates Fe transport in vascular tissues by repressing the expression of *NICOTIANAMINE SYNTHASE 4* (*NAS4*) and *FERRIC REDUCTASE OXIDASE 3* (*FRO3*) (Long et al., 2010). In contrast to the repressive role of PYE on *NAS4*, MYB72 and MYB10 directly induce *NAS4* transcription, acting antagonistically with PYE to co‐regulate Fe transport in the phloem, thereby maintaining Fe homeostasis in plants (Palmer et al., 2013). Post‐transcriptionally, the E3 ubiquitin ligases BTS and its homologs BTS LIKE1/2 (BTSL1/2) serve as negative regulators by targeting PYE and FIT pathway, respectively, thereby maintaining Fe homeostasis (Hindt et al., 2017; Liang et al., 2017; Rodríguez‐Celma et al., 2019). Additionally, IRON MAN/FE‐UPTAKE‐INDUCING PEPTIDE (IMA/FEP) peptides enhance Fe‐deficiency tolerance by stimulating the expression of *bHLH038*/39 (Grillet et al., 2018; Hirayama et al., 2018).

The NAC transcription factor family is one of the largest and most functionally diverse families in plants (Riechmann et al., 2000). Numerous NAC members have been implicated in regulating responses to a wide range of biotic and abiotic stresses (Bian et al., 2020; He et al., 2005; Kacprzak et al., 2020; Masri & Kiss, 2023), including flooding (Bui et al., 2020; Meng et al., 2020), extreme temperatures (Alshareef et al., 2022; Guo et al., 2015), drought and salinity (Hu et al., 2008). Several NAC transcription factors also function as master switches in cell wall biosynthesis (Hussey et al., 2013). For instance, ANAC050 enhances aluminum (Al) tolerance by coordinating organic acid secretion and hemicellulose deposition (Tao et al., 2025), and promotes Fe reutilization from the cell wall under Fe deficiency by modulating abscisic acid accumulation (Huang et al., 2025). Similarly, ANAC017 participates in diverse biological processes, including leaf senescence (Broda et al., 2021), mitochondrial retrograde signaling (Zhu et al., 2023), the mitochondrial dysfunction response (Pascual et al., 2021) and salt stress tolerance (Tao et al., 2023). In addition, ANAC017 contributes to Al tolerance by regulating cell wall‐modifying genes (Tao et al., 2022), while ANAC070 acts upstream of the ANAC017‐*XTH31* module to negatively regulate xyloglucan abundance and Al‐binding capacity in the cell wall (Li et al., 2024). Despite these advances, whether and how *ANAC017* contributes to Fe nutrition remains unknown. In this study, we demonstrate that ANAC017 is indispensable for maintaining Fe homeostasis in *Arabidopsis*, and loss of *ANAC017* function impairs the remobilization of cell wall Fe. Furthermore, we show that ANAC017 acts downstream of the core Fe regulator bHLH121/URI and directly regulates the expression of *MAT1*/*SAM1*, a gene encoding *S*‐adenosylmethionine synthetase. Given that SAM is a key precursor for the biosynthesis of both ethylene and NA, our findings suggest that ANAC017 may modulate ethylene biosynthesis and NA‐mediated long‐distance Fe transport via *MAT1/SAM1*, thereby contributing to Fe homeostasis in *Arabidopsis*.

## RESULTS

### ANAC017 is involved in the homeostasis of multiple mineral elements in *Arabidopsis*

To determine whether ANAC017 contributes to mineral nutrient homeostasis, we profiled the ionomic composition of *anac017* mutant roots under nutrient‐sufficient conditions. With the exception of boron (B), the *anac017* mutants exhibited significantly lower concentrations of all analyzed elements than wild‐type plants, including sodium (Na), magnesium (Mg), phosphorus (P), sulfur (S), potassium (K), calcium (Ca), manganese (Mn), Fe, copper (Cu), zinc (Zn), and molybdenum (Mo) (Figure 1). These widespread reductions in elemental concentrations suggest that ANAC017 contributes to the maintenance of ionomic homeostasis in *Arabidopsis*.

**Figure 1:**
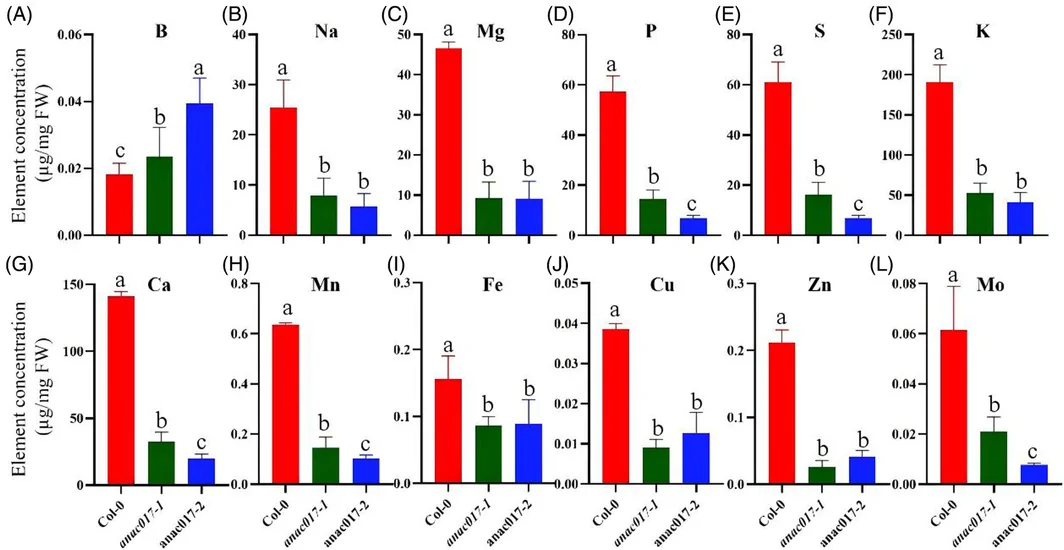
*ANAC017* is critical for ionomic homeostasis in *Arabidopsis* roots. B (A), Na (B), Mg (C), P (D), S (E), K (F), Ca (G), Mn (H), Fe (I), Cu (J), Zn (K), and Mo (L) concentrations in roots of wild‐type *Arabidopsis* (Col‐0) and the *anac017* mutants under standard nutrient conditions. Agar plate culture was used in the experiment. After 10 days of seedling growth, the root samples were collected for digestion and determination of related elements. Data are presented as mean ± SD of *n* = 3 biological replicates from one representative experiment. Similar results were obtained in at least two independent experiments. Significant differences were determined by one‐way ANOVA followed by Tukey's post hoc test (*P* < 0.05).

### The expression of *ANAC017* is preferentially induced by Fe deficiency

To explore the involvement of *ANAC017* in abiotic stress responses, we examined its transcript abundance in roots subjected to B, Mg, P, S, K, Ca, Fe, Zn, Cu, Mo, or Mn deficiency, as well as salt stress (NaCl). Among these treatments, Fe deficiency elicited the most pronounced induction of *ANAC017* expression (Figure 2A), suggesting a potential role for *ANAC017* in the Fe‐deficiency response. To determine whether *ANAC017* is preferentially involved in Fe deficiency, we next investigated the responses of wild‐type and *anac017* plants to Cu, Mn, or Zn deficiency. In contrast to the pronounced sensitivity of *anac017* mutants to Fe deficiency, no obvious hypersensitivity to Cu, Mn, or Zn deficiency was observed (Figure S1A). We further examined the expression of representative genes encoding Cu transporters (COPT1/2/3/4/5/6), natural resistance‐associated macrophage proteins (NRAMP1/2/3/4, NRAMP6), and Zn transporters (ZIP1–3; Heavy Metal ATPase, HMA2, HMA4). Although several genes (e.g., *COPT3*, *COPT6*, and *NRAMP6*) showed modest differential expression, the degree of dysregulation was substantially less pronounced than that observed in the Fe‐responsive network (Figure S1B–Q). To further substantiate this observation, we reanalyzed a publicly available RNA‐seq dataset (Broda et al., 2021). Gene Ontology (GO) enrichment analysis revealed significant enrichment of terms associated with ‘Fe homeostasis’ and ‘cellular response to Fe starvation’ (Figure 2C), providing independent evidence for the involvement of ANAC017 in Fe‐related processes. Moreover, the *anac017* mutant exhibited strong upregulation of *bHLH38* and *bHLH39* (Figure 2B), two central regulators of Fe acquisition and redistribution conserved across *Arabidopsis*, tomato, rice, soybean and maize (Kurt & Filiz, 2018). Taken together, these results support a prominent role for ANAC017 in the adaptive response to Fe deficiency.

**Figure 2:**
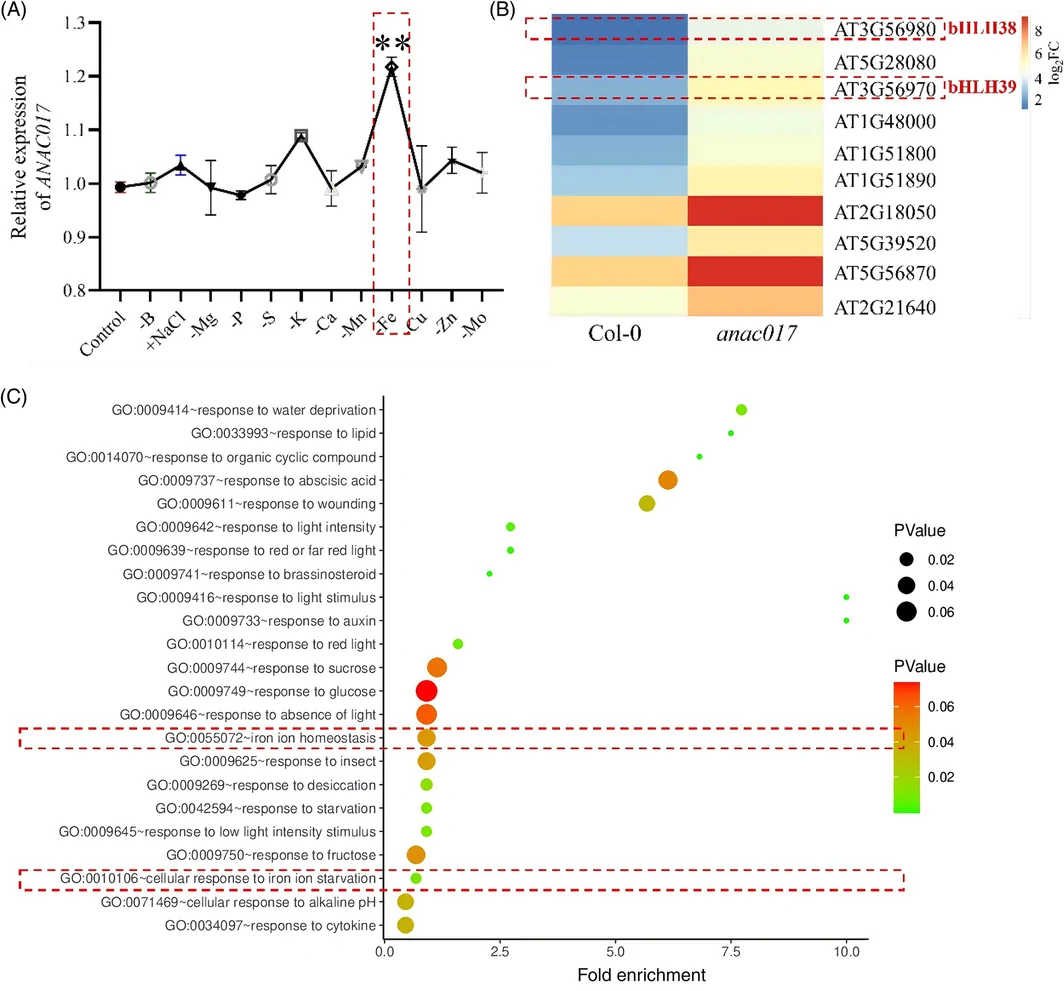
*ANAC017* is required for the response to Fe deficiency. (A) Relative expression of *ANAC017* under various nutrient deficiency. Col‐0 seedlings were grown under Fe‐sufficient condition for 14 days and then subjected to various stress treatments for 7 days. Root tissues were harvested for RNA extraction and qPCR analysis. The expression of *ANAC017* under control condition was normalized as ‘1’. Data are presented as mean ± SD (*n* = 3 biological replicates). Significant differences were determined by one‐way ANOVA followed by Tukey's post hoc test (**P < 0.01). (B) Heatmap of the top 10 upregulated genes in *anac017* mutants compared with Col‐0 plants in response to abiotic stress. Data were obtained from Broda et al. (2021). Red rectangles highlight Fe‐related genes. (C) Gene Ontology (GO) enrichment analysis between Col‐0 and *anac017* mutants in response to several abiotic stresses. Red rectangles highlight Fe‐related biological processes.

### Fe deficiency upregulates *ANAC017* expression and protein abundance

To further characterize the role of *ANAC017* in the Fe‐deficiency response, we examined its transcriptional and translational dynamics under ‐Fe conditions. A short‐term time‐course assay revealed that *ANAC017* transcript levels in roots increased dramatically within 0.5 h of Fe deprivation (Figure 3A), indicating rapid transcriptional activation in response to Fe deficiency. Consistent with this response, immunoblot analysis of plants expressing *ANAC017‐GFP* showed that ANAC017‐GFP abundance markedly increased under Fe‐deficient conditions (Figure 3B). GUS staining of *ProANAC017::GUS* reporter seedlings further confirmed the strong induction of *ANAC017* by Fe deficiency (Figure 3C). Together, these findings demonstrate that ANAC017 is rapidly and robustly induced by Fe deprivation at both transcript and protein levels.

**Figure 3:**
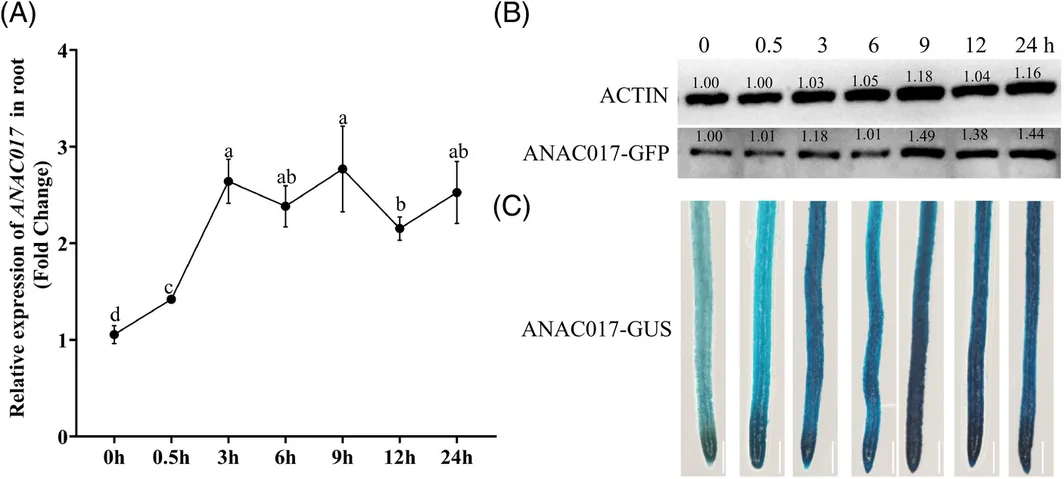
Expression patterns of ANAC017 at the transcript and protein levels under Fe deficiency. (A) Time‐course response of *ANAC017* transcript levels to Fe deficiency (0, 0.5, 3, 6, 9, 12, and 24 h). Data are presented as mean ± SD of *n* = 4 biological replicates from one representative experiment. The experiment was independently repeated three times with similar results. Significant differences were determined by one‐way ANOVA followed by Tukey's post hoc test (*P* < 0.05). (B) Time‐course response of ANAC017 protein accumulation under Fe deficiency. Four‐week‐old seedlings expressing *ANAC017‐GFP* were transferred to Fe‐deficient nutrient solution and harvested at the indicated time points (0, 0.5, 3, 6, 9, 12, and 24 h). Total proteins were extracted for immunoblot analyses. (C) Time‐course analysis of *ANAC017* promoter activity in response to Fe deficiency. Four‐week‐old *ProANAC017::GUS* transgenic seedlings were subjected to Fe deficiency and harvested at the indicated time points (0, 0.5, 3, 6, 9, 12, and 24 h) for GUS staining. Scale bars = 100 μm.

### Lack of functional ANAC017 results in hypersensitivity to Fe deficiency

To determine whether *ANAC017* contributes to Fe‐deficiency tolerance, we compared the growth of wild‐type and *anac017* seedlings transferred to Fe‐sufficient (+Fe) and Fe‐deficient (−Fe) media. Under +Fe conditions, no obvious phenotypic differences were observed between wild‐type and *anac017* plants. In contrast, under −Fe conditions, *anac017* mutants displayed more pronounced growth inhibition than wild type (Figure 4A), as reflected by significantly shorter primary roots (Figure 4B). These results indicate that lack of functional ANAC017 compromises tolerance to Fe deficiency. A hydroponic assay further supported this observation; under Fe‐limited conditions, young leaves of *anac017* plants exhibited more severe chlorosis and significantly lower SPAD values than the wild type (Figure 4C,D). Conversely, *ANAC017*‐overexpression (*ANAC017*‐OX) and the complementation (*ANAC017*‐COM) lines displayed enhanced tolerance to Fe deficiency (Figures S2 and S3), further supporting a role for ANAC017 in maintaining Fe homeostasis under Fe‐limiting conditions.

**Figure 4:**
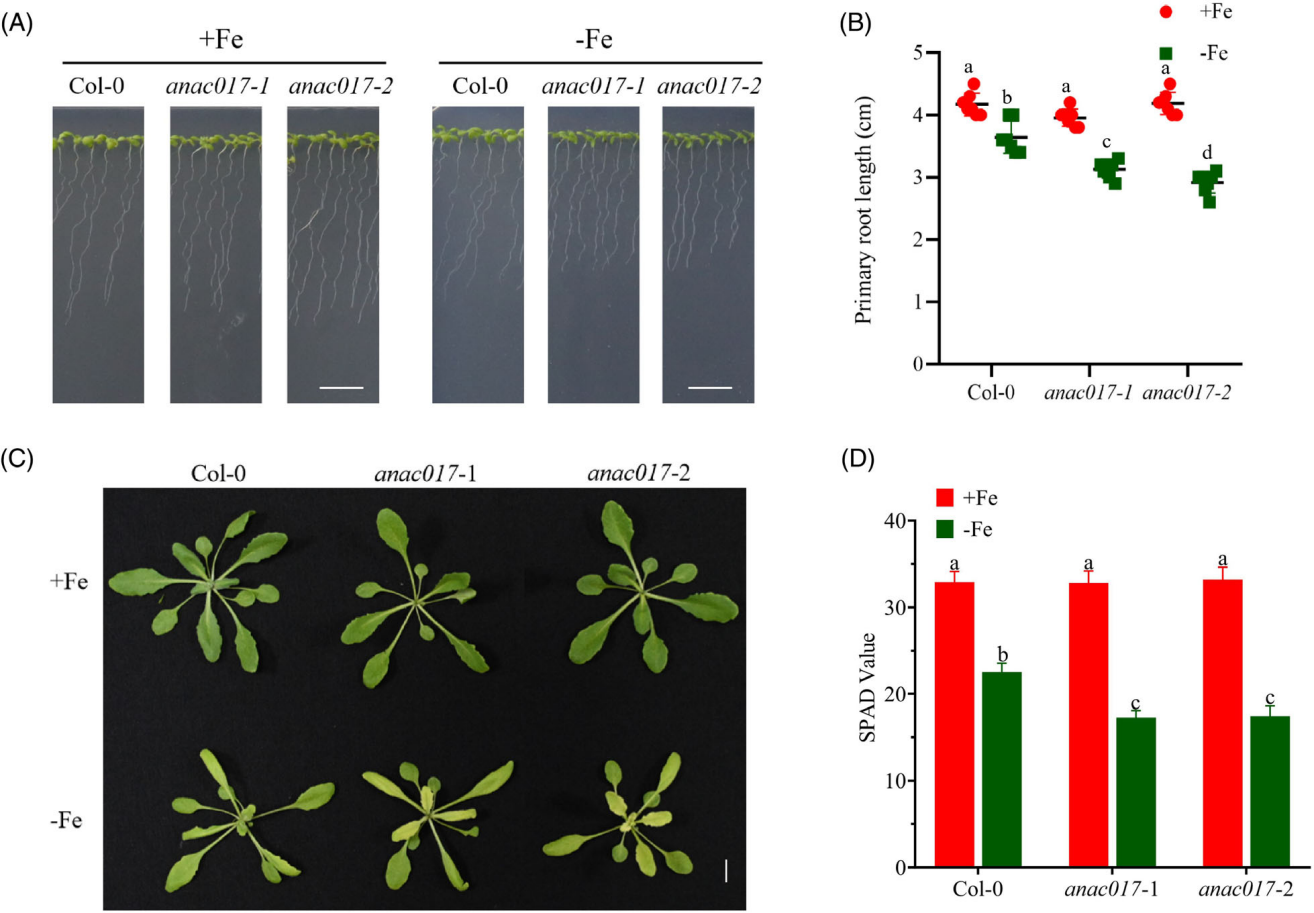
*anac017* mutants exhibit sensitivity to Fe deficiency. (A) Phenotypes and (B) primary root elongation of wild‐type Col‐0 and *anac017* mutant seedlings grown on agar plates under Fe‐sufficient (+Fe) or Fe‐deficient (−Fe) conditions (*n* = 7 individual plants). Seedlings were first germinated and grown on 1/2 MS medium until primary roots reached approximately 1 cm in length, then transferred to +Fe (control) or −Fe media and grown for an additional 7 days. Scale bar = 1 cm. (C) Phenotypes and (D) SPAD values (reflecting relative chlorophyll content) of Col‐0 and *anac017* plants in hydroponic culture under +Fe and −Fe conditions. Four‐week‐old seedlings pre‐cultured in standard nutrient solution were selected for uniform growth and subsequently treated with +Fe or −Fe hydroponic solution for 7 days. Data are presented as mean ± SD (*n* = 10 individual plants). Scale bar = 1 cm. Representative data from three independent experiments are shown. For panels (B) and (D), significant differences were determined by two‐way ANOVA followed by Tukey's post hoc test (*P* < 0.05).

To elucidate the physiological basis of this phenotype, we quantified Fe accumulation in roots and shoots using ICP‐AES. Under +Fe conditions, Fe concentrations did not differ significantly between *anac017* mutants and wild‐type plants. However, under −Fe conditions, Fe levels were markedly reduced in both roots and shoots of *anac017* mutants (Figure 5A,B), whereas *ANAC017*‐OX plants accumulated higher Fe levels than the wild type (Figure S2E,F). We next assessed the activity of FCR, a hallmark of Fe‐deficiency response. Under +Fe conditions, FCR activity was comparable across genotypes (Figure 5C; Figure S2G). Under −Fe conditions, FCR activity was increased in all seedlings but was significantly higher in *anac017* mutants than in wild‐type plants (Figure 5C). Conversely, *ANAC017‐*OX plants exhibited reduced FCR activity compared to the wild type (Figure S2G). These results collectively indicate that ANAC017 modulates Fe‐deficiency responses and is required for maintaining Fe homeostasis under ‐Fe conditions.

**Figure 5:**
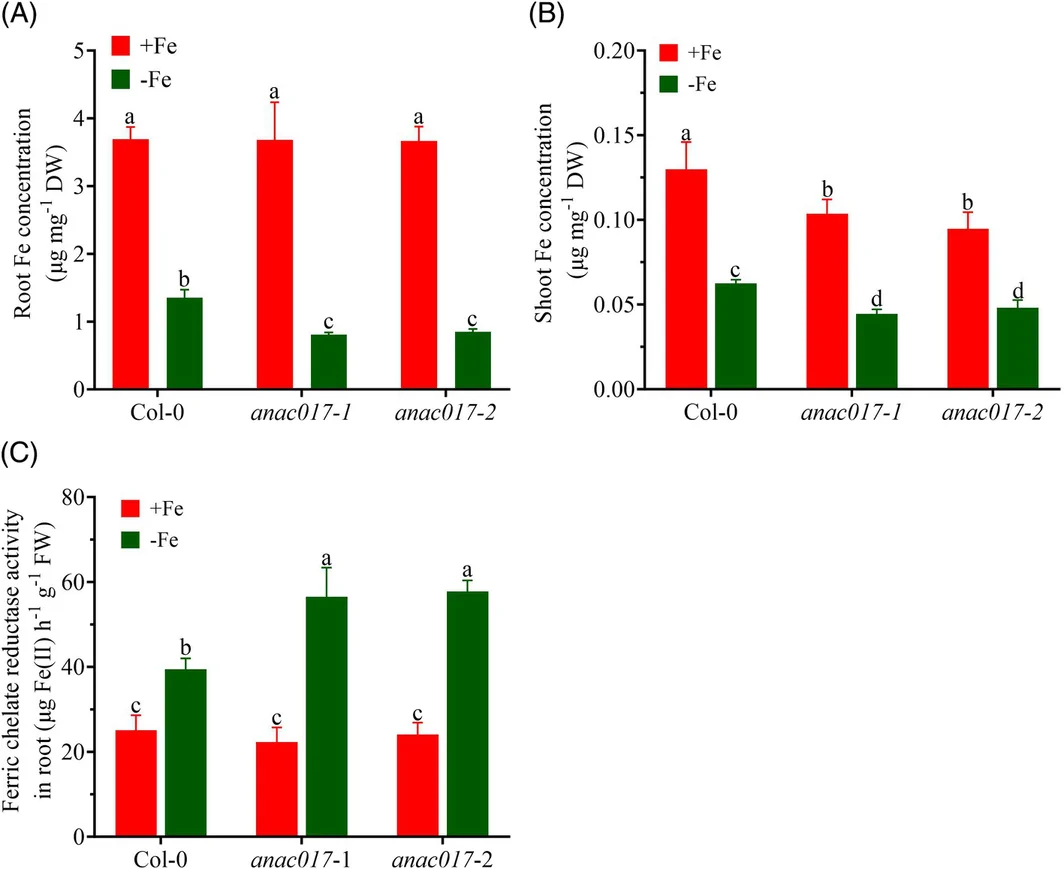
*ANAC017* affects Fe uptake and transport in *Arabidopsis*. (A) Root Fe content, (B) shoot Fe content, and (C) root ferric chelate reductase (FCR) activity in Col‐0 and *anac017* mutants grown under Fe‐sufficient (+Fe) or Fe‐deficient (−Fe) conditions. Four‐week‐old seedlings of Col‐0 and *anac017* mutants were transferred to nutrient solutions with or without Fe and cultured for 7 days prior to sampling and physiological measurements. Data are presented as mean ± SD (*n* = 4 individual plants). Representative data from two independent experiments are shown. Significant differences were determined by two‐way ANOVA followed by Tukey's post hoc test (*P* < 0.05).

### Lack of functional ANAC017 leads to increased Fe accumulation in root cell wall

Cell wall‐bound Fe represents a major apoplastic Fe pool and a potential source for remobilization under Fe limitation. To determine whether ANAC017 affects cell wall‐associated Fe, we quantified cell wall composition and Fe partitioning in roots. Under Fe‐deficient conditions, *anac017* roots accumulated significantly more Fe in the total cell wall, and specifically in the hemicellulose fraction (Figure 6A,B). In contrast, total hemicellulosic sugar content did not differ significantly across genotypes (Figure 6C), suggesting that the increased Fe accumulation in these fractions was not attributable to altered hemicellulose abundance. Furthermore, given the reduced total Fe content observed in the *anac017* mutants under Fe‐deficient conditions (Figure 5A), these findings imply that ANAC017 is likely involved in the remobilization of cell wall‐associated Fe, thereby contributing to internal Fe allocation.

**Figure 6:**
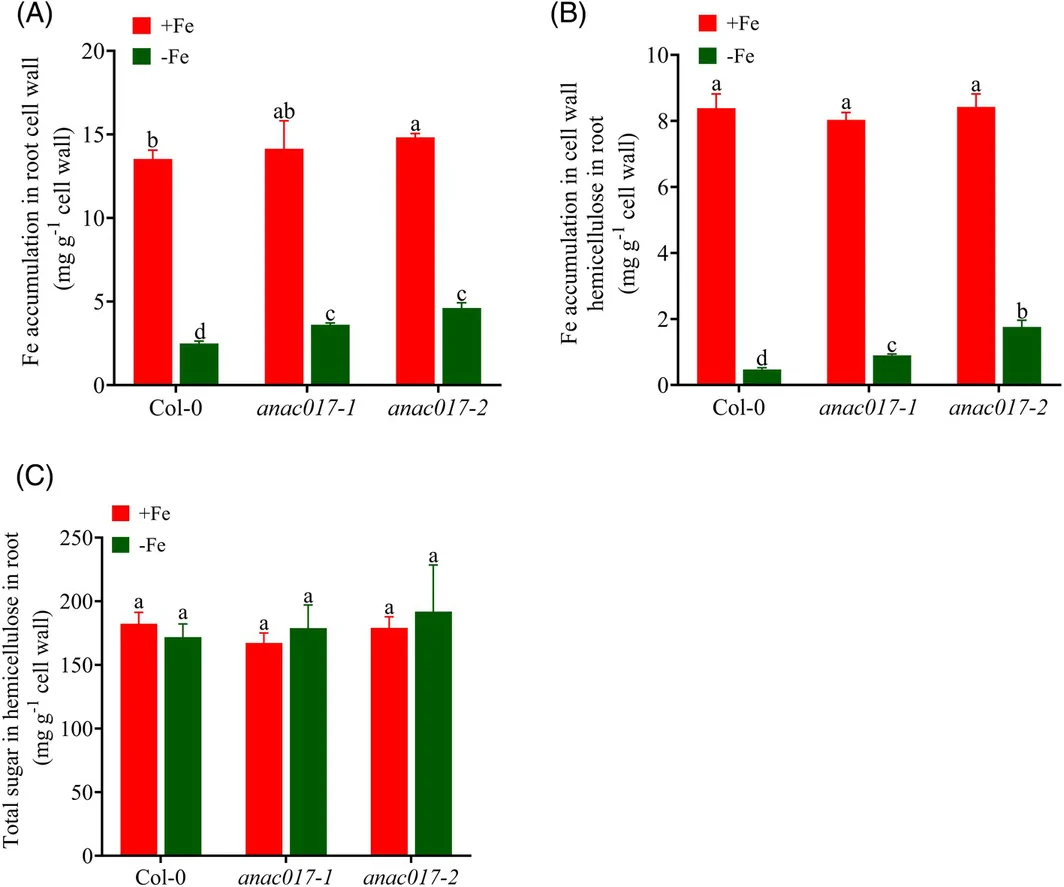
*ANAC017* affects Fe remobilization from the cell wall in *Arabidopsis*. Contents of (A) cell wall‐bound Fe, (B) hemicellulose‐bound Fe and (C) total hemicellulose sugars in roots of Col‐0 and *anac017* mutants under Fe‐sufficient (+Fe) or Fe‐deficient (−Fe) conditions. Four‐week‐old seedlings were transferred to nutrient solutions with or without Fe for 7 days prior to analysis. Data are presented as mean ± SD (*n* = 4 individual plants), and the experiment was repeated twice with similar results. Significant differences were determined by two‐way ANOVA followed by Tukey's post hoc test (*P* < 0.05).

### Loss of ANAC017 function dysregulates the transcriptional response to Fe deficiency

Because Fe deficiency triggers extensive transcriptional reprogramming, we examined whether the disruption of *ANAC017* alters canonical Fe‐responsive gene expression. Quantitative real‐time PCR (qRT‐PCR) profiling revealed widespread dysregulation of key components within the Fe network in *anac017* mutants (Table 1). Under Fe‐deficient conditions, the expression of *bHLH38*, *bHLH39*, *FIT*, and *PYE* was markedly upregulated in *anac017* mutants compared with the wild type, whereas *MYB10* was strongly down‐regulated. Consistent with these transcriptional shifts, the expression of Fe‐uptake markers *IRT1* and *FRO2* was hyper‐induced in *anac017* mutants under −Fe stress. Further analysis of long‐distance transport and chelation genes revealed genotype‐specific responses: The expression of *NAS1*, *FRD3*, and *YSL2* was similar to the wild type under +Fe condition, whereas *YSL2* showed strong induction in *anac017* under −Fe condition. Notably, under +Fe or Fe‐limiting conditions, the expression of *XTH31*, a gene involved in cell wall hemicellulose synthesis and modification, was significantly suppressed in the *anac017* mutants. Taken together, these results demonstrate that the disruption of *ANAC017* profoundly perturbs the Fe‐deficiency transcriptional program, implicating ANAC017 as a critical role required for appropriate regulation of Fe uptake, transport, and apoplastic mobilization.

**Table 1:** Comparison of the expression levels of Fe deficiency‐responsive genes between wild type (Col‐0) and *anac017* mutants

Gene	+Fe			−Fe		
	Col‐0	*anac017‐1*	*anac017‐2*	Col‐0	*anac017‐1*	*anac017‐2*
*bHLH121/URI*	1.016 ± 0.080 a	1.145 ± 0.144 a	1.155 ± 0.102 a	1.286 ± 0.165 a	0.992 ± 0.188 b	1.131 ± 0.247 ab
*bHLH38*	1.018 ± 0.072 f	2.164 ± 0.327 d	1.422 ± 0.140 e	44.354 ± 3.497 c	163.676 ± 19.883 a	131.252 ± 18.634 b
*bHLH39*	1.022 ± 0.098 d	3.510 ± 0.496 c	5.925 ± 1.319 b	2.681 ± 0.513 c	6.095 ± 2.486 b	18.177 ± 2.595 a
*FIT*	1.064 ± 0.093 c	1.139 ± 0.088 c	1.085 ± 0.136 c	3.998 ± 0.636 b	7.621 ± 1.162 a	6.645 ± 1.183 a
*PYE*	1.002 ± 0.0670 a	0.725 ± 0.066 c	0.864 ± 0.058 b	0.265 ± 0.089 e	1.015 ± 0.148 a	0.538 ± 0.071 d
*IMA1*	1.005 ± 0.116 c	1.378 ± 0.143 b	1.361 ± 0.111 b	1.605 ± 0.390 a	1.962 ± 0.239 a	1.690 ± 0.207 a
*MYB10*	1.044 ± 0.334 c	1.035 ± 0.312 c	1.038 ± 0.429 c	9.043 ± 1.165 a	5.983 ± 2.226 b	4.603 ± 0.975 b
*IRT1*	1.067 ± 0.210 c	0.777 ± 0.325 cd	0.591 ± 0.109 d	308.121 ± 70.985 b	550.106 ± 65.426 a	522.692 ± 32.096 a
*FRO2*	1.015 ± 0.034 d	0.702 ± 0.079 e	0.809 ± 0.067 e	34.183 ± 3.35 c	87.393 ± 4.845 a	66.765 ± 7.146 b
*FRD3*	1.011 ± 0.086 a	0.725 ± 0.081 b	1.011 ± 0.094 a	0.35 ± 0.040 c	0.296 ± 0.016 c	0.337 ± 0.043 c
*YSL2*	1.017 ± 0.079 b	1.241 ± 0.106 b	1.215 ± 0.156 b	0.711 ± 0.105 c	1.683 ± 0.163 a	1.584 ± 0.227 a
*NAS1*	1.003 ± 0.126 a	0.908 ± 0.051 a	0.888 ± 0.093 a	1.052 ± 0.200 a	0.353 ± 0.017 b	0.330 ± 0.043 b
*NAS4*	1.004 ± 0.058 c	1.525 ± 0.059 b	1.426 ± 0.206 b	1.949 ± 0.0145 a	1.466 ± 0.263 b	1.159 ± 0.264 c
*NAS2*	1.024 ± 0.240 b	1.143 ± 0.192 b	1.009 ± 0.279 b	1.681 ± 0.381 a	1.031 ± 0.303 b	0.9449 ± 0.071 b
*XTH31*	1.020 ± 0.033 a	0.609 ± 0.081 b	0.618 ± 0.060 b	0.310 ± 0.037 c	0.255 ± 0.040 cd	0.213 ± 0.021 d

Four‐week‐old wild‐type and *anac017* mutant plants were subjected to either control or Fe‐deficient conditions for 7 days. RNA was extracted from root tissues. *ACTIN* was used as an internal control. Data are presented as mean ± SD (*n* = 4 biological replicates). Significant differences were determined by two‐way ANOVA followed by Tukey's post hoc test (*P* < 0.05).

### ANAC017 does not directly interact with core components of the Fe regulatory network

The pronounced alterations in transcript levels of key Fe homeostasis regulators, such as *bHLH38* and *bHLH39*, observed in *anac017* mutants under Fe deficiency (Figure 2B and Table 1) prompted us to examine whether ANAC017 physically interacts with known Fe‐associated transcription factors and regulatory proteins, including bHLH34, bHLH104, bHLH105, bHLH100, WRKY46, FIT, PYE, MYB10, and MYB72. Given the previous observation that ANAC017 exhibits transcriptional autoactivation (Tao et al., 2022), we employed a yeast two‐hybrid assay to assess protein–protein interactions while suppressing autoactivation effects. Of all the combinations tested, ANAC017 failed to interact with any of the examined proteins in yeast (Figure S4). These results indicate that ANAC017 does not directly engage with core components of the established bHLH‐centered Fe regulatory module, and instead likely influences Fe homeostasis through an indirect or parallel regulatory mechanism.

### The expression of *ANAC017* is regulated by bHLH121/URI in response to Fe deficiency

To determine whether *ANAC017* is functionally embedded within the bHLH‐centered Fe regulatory network, we first employed a yeast‐based screening system to identify its upstream interactors. This screen revealed interactions between ANAC017 and two Fe‐associated proteins: bHLH121/URI and MYB10 (Figure 7A,B). Notably, bHLH121/URI, a master transcription factor acting upstream of multiple Fe homeostasis pathways (Lei et al., 2020), was found to directly regulate *ANAC017* expression (Figure 7A). We next validated this regulatory relationship using a luciferase (LUC) reporter assay. Co‐transformation of the bHLH121/URI effector construct with the *ANAC017* promoter‐driven reporter significantly enhanced LUC activity compared with the empty vector control (Figure 7C,D), confirming that *ANAC017* is a transcriptional target of bHLH121/URI. Expression analysis of *ANAC017* in wild‐type and *bhlh121* mutant plants under Fe stress conditions revealed that the Fe‐deficiency‐induced expression of *ANAC017* is dependent on bHLH121 (Figure 7E).

**Figure 7:**
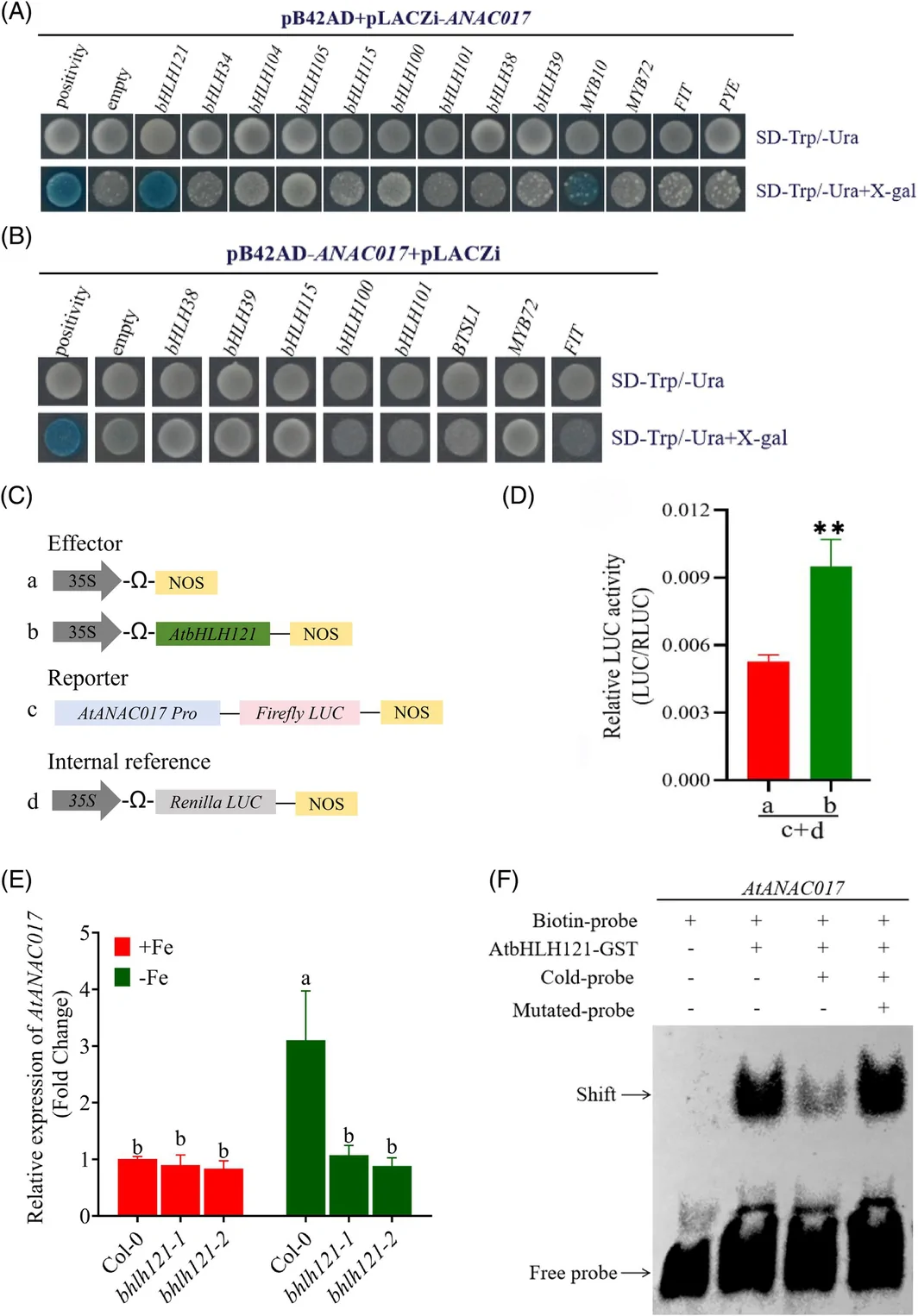
Regulation of *ANAC017* expression by the transcription factor bHLH121/URI. (A) Yeast one‐hybrid analysis of the interaction between key Fe homeostasis‐related transcription factors and the *ANAC017* promoter. The experiment was repeated at least three times. (B) Yeast one‐hybrid analysis of the interaction between ANAC017 and the promoters of key Fe homeostasis‐related transcription factors. The experiment was repeated at least three times. (C) Schematic representation of the reporter construct (*ANAC017pro::LUC*), the effector plasmids, and the internal transfection control used in the dual‐luciferase assay. a: Empty vector control; b: *AtbHLH121* expression vector; c: *AtANAC017* expression vector; d: *AtANAC017* promoter::Firefly luciferase reporter; e: Internal reference construct, *35S* promoter::Renilla luciferase internal control. (D) Relative LUC activity of the *ANAC017pro::LUC* activity with co‐expression of 35S‐*bHLH121/URI* and 35S‐*ANAC017*. 35S‐empty vector was used as the effector plasmid control. In each treatment, the letters a–e correspond to the components of the firefly luciferase activity detection system shown in (C). The values shown are the means, and error bars represent ± SD (*n* = 3 biological replicates), and the experiment was repeated twice with similar results. Significant difference was determined by Student's *t*‐test (*P* < 0.01). (E) Expression analysis of *ANAC017* in Col‐0 and *bhlh121* mutants under control and Fe‐deficient conditions. Four‐week‐old seedlings were grown under +Fe or ‐Fe conditions for 7 days. Root tissues were harvested, and transcript levels of *ANAC017* were examined by qPCR using *AtACTIN* as the internal reference gene. Data are presented as means ± SD (*n* = 4 individual plants), and the experiment was repeated three times. Significant differences were determined by two‐way ANOVA followed by Tukey's post hoc test (*P* < 0.05). (F) EMSA analysis showing the direct binding of bHLH121 protein to the *ANAC017* promoter. Purified AtbHLH121‐GST was incubated with biotin‐labeled probes. For the competition assay, the unlabeled specific competitor (Cold‐probe) and the mutated probe (Mutated‐probe) were added at the same concentration and equal volume as the biotin‐labeled probe (1:1 ratio). Lanes 1–4 indicate: Biotin‐probe only (negative control); Biotin‐probe + AtbHLH121‐GST protein (binding assay); Biotin‐probe + AtbHLH121‐GST + Cold‐probe (competition assay); Biotin‐probe + AtbHLH121‐GST + Mutated‐probe (mutation assay). ‘Shift’ indicates the protein‐DNA complex, and ‘Free probe’ indicates unbound DNA. The experiment was repeated twice with similar results.

To further confirm the direct binding of bHLH121 to *ANAC017* promoter, the electrophoretic mobility shift assay (EMSA) was performed. Since most bHLHs primarily bind E/G‐box cis‐acting elements to regulate the expression of their target genes (Zhao et al., 2024), we analyzed the *ANAC017* promoter sequence and identified 9 putative E/G‐box binding sites. Through EMSA assays, we demonstrated that bHLH121 directly binds to the promoter of *ANAC017* (Figure 7F).

To assess the functional relevance of the bHLH121/URI‐*ANAC017* regulatory module under Fe‐deficient conditions, we overexpressed *ANAC017* in the *bhlh121/uri* mutant. Phenotypic analysis showed that *ANAC017* overexpression (*ANAC017*‐OX/*bhlh121*) rescued the Fe sensitivity of the *bhlh121/uri* mutant (Figure 8A,B), as evidenced by increased primary root length and SPAD values (Figure 8C,D), as well as increased Fe accumulation in both roots and shoots in complementation lines (Figure 8E,F). Together, these findings demonstrate that bHLH121/URI acts upstream of *ANAC017* and is a critical component of the Fe‐deficiency regulatory pathway.

**Figure 8:**
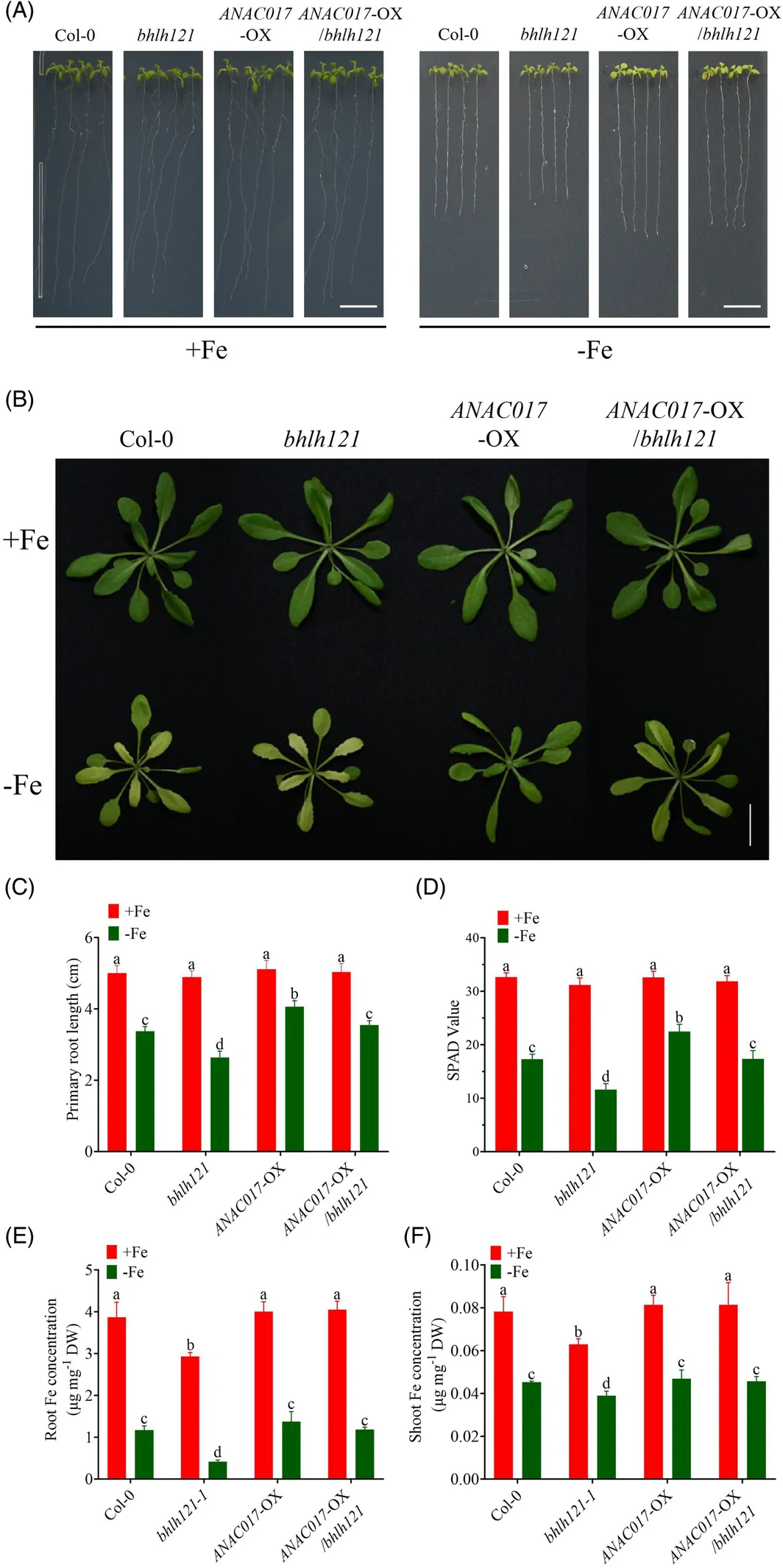
Overexpression of *ANAC017* partially rescues the Fe‐deficiency sensitivity in *bhlh121* mutant. (A, B) Phenotypes of wild‐type (Col‐0), *bbhlh121* mutant, *ANAC017*‐OX, and *ANAC017*‐OX/*bhlh121* lines under Fe‐sufficient and Fe‐deficient conditions in plate assays (A) and hydroponic culture (B) For plate assay, seedlings were transferred to the corresponding media when the primary roots had grown to ≥1.2 cm, and grown for 7 days. In the hydroponic assay, 4‐week‐old seedlings were subjected to treatment. Scale bar = 2 cm. (C) Root length of seedlings measured after the 7‐day treatment on plate media. Data are presented as means ± SD (*n* = 6 individual plants), and the experiment was repeated three times. (D) Leaf SPAD values measured after the 7‐day hydroponic treatment. Data are presented as means ± SD (*n* = 8 individual plants). (E, F) Fe content in roots (E) and shoots (F) as determined by ICP‐AES following sample digestion after the 7‐day hydroponic treatment. Data are presented as means ± SD (*n* = 4 individual plants). For panels C–F, the experiment was repeated twice with similar results, and significant differences were determined by two‐way ANOVA followed by Tukey's post hoc test (*P* < 0.05).

### ANAC017 regulates Fe homeostasis in *Arabidopsis* by modulating the downstream gene *MAT1/SMA1*

To dissect the downstream signaling of ANAC017, we performed a yeast one‐hybrid assay to identify potential transcriptional targets of ANAC017. Interestingly, yeast one‐hybrid assays showed that ANAC017 interacts with *MAT1*/*SAM1*, which encodes methionine adenosyltransferase/*S*‐adenosylmethionine synthetase, suggesting that ANAC017 may directly regulate *MAT1*/*SAM1* expression (Figure 9A). Transcript abundance analysis showed that loss of *ANAC017* function significantly altered the expression levels of *MAT1*/*SAM1* (Figure 9B), and this regulatory effect was further supported by dual‐luciferase reporter assays (Figure 9C,D).

**Figure 9:**
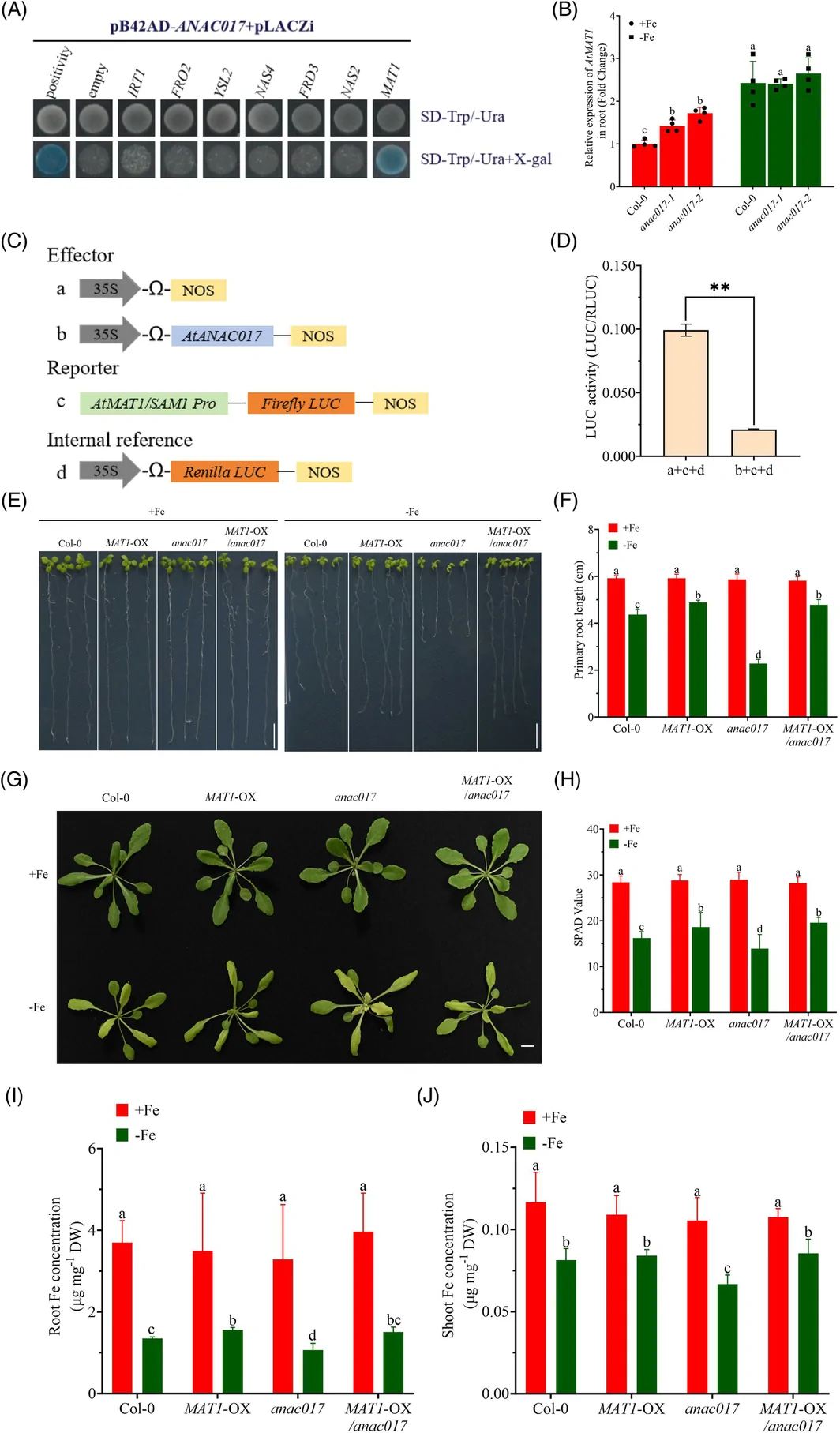
ANAC017 regulates *Arabidopsis* Fe homeostasis by regulating *MAT1/SMA1*. (A) Yeast one‐hybrid assay showing the binding of ANAC017 to the *MAT1/SMA1* promoter. (B) Expression analysis of *MAT1/SAM1* in roots of Col‐0 and *anac017* mutants under control (+Fe) and Fe‐deficient (−Fe) conditions. Data are presented as means ± SD (*n* = 4 individual plants). Significant differences were determined by two‐way ANOVA followed by Tukey's post hoc test (*P* < 0.05). (C) Dual‐luciferase reporter assay for the interaction between ANAC017 protein and the *MAT1/SAM1* promoter. a: Empty vector control; b: AtANAC017 expression vector; c: *AtMAT1/SAM1* promoter::Firefly luciferase reporter; d: Internal reference construct. (D) Relative activity of the *ProAtMAT1/SAM1*::LUC reporter gene co‐transformed with the indicated effectors in rice protoplasts. Data are presented as means ± SD (*n* = 3 biological replicates). Significant difference was determined by Student's *t*‐test (*P* < 0.01). (E, G) Overexpression of *MAT1/SMA1* partially rescues the Fe‐deficiency hypersensitivity of the *anac017* mutant under Fe‐deficient conditions: (E) phenotype in plate assay. When the seedling primary roots reached ≥1.2 cm, they were transferred to the corresponding media and grown for 7 days; (G) phenotype in hydroponic culture. Approximately 4‐week‐old seedlings were transferred to various treatment conditions and allowed to grow for 7 days. Scale bar = 1 cm. (F, H–J) Quantification of phenotypic parameters: (F) root length (*n* = 6 individual plants); (H) leaf SPAD value (*n* = 10 individual plants); (I) root and (J) shoot Fe content (*n* = 5 individual plants). Data are presented as mean ± SD, and significant differences were determined by two‐way ANOVA followed by Tukey's post hoc test (*P* < 0.05). All the above experiments were independently repeated three times.

To further dissect the regulatory relationship between ANAC017 and MAT1/SAM1, we generated transgenic lines overexpressing *MAT1/SAM1* in the *anac017* mutant (*MAT1*‐OX/*anac017*). Phenotypic analyses revealed that *MAT1/SAM1* overexpression significantly rescued the Fe‐sensitive phenotype of the *anac017* mutant under Fe‐deficient conditions (Figure 9E,G), as evidenced by the improved primary root growth and increased SPAD values (Figure 9F,H). Moreover, Fe concentrations were significantly higher in both roots and shoots of the *MAT1*‐OX*/anac017* line compared with *anac017* mutants (Figure 9I,J). Collectively, these results support that ANAC017 functions upstream of *MAT1*/*SAM1*, highlighting the importance of the ANAC017‐*MAT1/SAM1* module in the *Arabidopsis* response to Fe deficiency.

## DISCUSSION

### ANAC017 is a transcription factor responsive to Fe deficiency

ANAC017 has been primarily characterized as a key regulator of mitochondrial retrograde signaling, contributing to endoplasmic reticulum protection (Fuchs et al., 2022) and delayed leaf senescence (Broda et al., 2021). However, its role in nutrient‐related stress responses has remained largely unexplored. Here, we reveal a previously unrecognized role for *ANAC017* in Fe homeostasis. The broad reduction in the concentrations of multiple mineral elements observed in the *anac017* mutants under normal growth conditions suggests that *ANAC017* contributes to the maintenance of basal ion balance in *Arabidopsis* roots (Figure 1). Notably, *ANAC017* expression was strongly induced by Fe deficiency at both transcript and protein levels (Figures 2 and 3), indicating that *ANAC017* is responsive to Fe status and may function as part of the Fe‐deficiency signaling network. Consistent with this notion, *anac017* mutants exhibit increased sensitivity to Fe limitation, characterized by reduced root elongation, chlorosis, diminished chlorophyll content, and increased FCR activity (Figures 4 and 5C). This hypersensitivity was accompanied by a dysregulated transcriptional response: Classical Fe‐deficiency marker genes, including *bHLH38/39*, *IRT1*, and *FRO2*, were hyper‐induced, whereas a subset of Fe transport and chelation genes (*MYB10*, *NAS1/2/4*) failed to be properly activated under Fe deficiency (Figure 2B and Table 1). These transcriptional perturbations were accompanied by substantially lower Fe content in both roots and shoots of the mutants under Fe‐limiting conditions (Figure 5A,B). Even under Fe‐sufficient conditions, shoot Fe accumulation remained compromised, suggesting a constitutive defect in Fe allocation. Additionally, the Fe transport‐related genes *FRD3* and *YSL2*, which are normally repressed under Fe deficiency, did not exhibit the expected expression patterns in *anac017* mutants, further supporting the role of *ANAC017* in coordinating iron homeostasis. Importantly, the Fe‐deficiency phenotypes of *anac017* were rescued by either transgenic overexpression or genomic complementation of *ANAC017*, while *ANAC017* overexpression alone enhanced tolerance to Fe deficiency (Figures S2 and S3). Together, these findings demonstrate that ANAC017 serves as a positive regulator of Fe homeostasis and contributes to plant adaptation to Fe‐limited environments.

### *ANAC017* is required for maintaining Fe homeostasis by facilitating the reutilization of hemicellulose‐associated Fe in the cell wall

The root cell wall is a major determinant of Fe allocation in *Arabidopsis*. Under Fe‐deficient conditions, the cell wall serves as the primary Fe reservoir (Bienfait, 1985; Lei et al., 2014), with hemicellulose representing a key Fe‐binding component critical for plant acclimation to Fe limitation (Liu et al., 2023; Zhu et al., 2016). A previous study by Tao et al. (2022) demonstrated that ANAC017 directly binds to the promoter of *XTH31*, which encodes an enzyme involved in xyloglucan accumulation and cell wall remodeling, and subsequently activates its transcription, thereby modulating hemicellulose composition and enhancing Al‐binding capacity under Al stress. Consistent with this regulatory role, loss of *ANAC017* function impairs *XTH31* expression and disrupts xyloglucan homeostasis. Moreover, Huang et al. (2025) reported that Fe deficiency represses the transcription of *XTH31*, and that *xth31* mutants are highly sensitive to Fe limitation. Consistently, our qRT‐PCR analysis revealed that *XTH31* expression was lower in *anac017* mutants than in Col‐0 under normal growth conditions and was further suppressed under Fe deficiency, reaching levels below those detected in Fe‐deprived wild‐type plants (Table 1).

To investigate whether ANAC017 regulates Fe homeostasis through modulating the cell wall‐bound Fe pool, we examined Fe distribution within the root cell wall, with particular emphasis on the hemicellulose fraction. Under Fe deficiency, the *anac017* mutant exhibited markedly higher Fe retention in both total root cell wall and hemicellulose fraction (Figure 6A,B), despite showing no significant alteration in overall hemicellulose content (Figure 6C). Strikingly, total Fe content in both roots and shoots of the *anac017* mutants was substantially lower than that of wild‐type plants under the same conditions (Figure 5A). These contrasting patterns—comparable hemicellulose levels, elevated wall‐bound and hemicellulose‐associated Fe, but reduced total tissue Fe—led us to infer that loss of *ANAC017* function may impede the reutilization of Fe stored in hemicellulose. At first glance, this observation appears to contrast with the findings by Zhu et al. (2012) and Tao et al. (2022) under Al stress. However, it should be noted that the experimental conditions differ fundamentally between these studies. While previous research focused on toxicity induced by a non‐essential metal (excess Al), the present study examines the deficiency of an essential micronutrient (Fe). These contrasting stress conditions likely recruit ANAC017 into distinct regulatory pathways, resulting in different downstream responses. Furthermore, cell wall modification is a complex process mediated by multiple enzymes. In *Arabidopsis*, 33 XTH family members have been identified (Yokoyama & Nishitani, 2001), which exhibit diverse expression patterns in response to various environmental stimuli and may be regulated by specific signals to perform distinct functions. For example, XTH14, XTH15, and XTH17 have been shown to play important roles in hemicellulose content regulation (Zhu et al., 2012, 2013, 2014). Specifically, loss of *XTH15* function reduces cell wall Al accumulation (Zhu et al., 2013). Moreover, XTH17 has been shown to bind with XTH31 both *in vitro* and *in vivo* to form a dimer that regulates cell wall Al binding, and the *xth17* mutant exhibits a phenotype very similar to that of *xth31* (Zhu et al., 2014). Given the functional redundancy of XTH family members in hemicellulose modification, we speculate that ANAC017 may target other *XTH* genes in addition to *XTH31*, thereby affecting hemicellulose modification and/or content in the cell wall. This regulatory cascade may also exhibit stress‐specific preferences. Under Fe‐deficient conditions, ANAC017 might regulate Fe homeostasis not only via *XTH31* but also potentially through other *XTH* genes to modulate Fe‐deficiency tolerance. However, this hypothesis requires further investigation.

### *ANAC017* defines a novel branch downstream of bHLH121/URI in the Fe regulatory network

Fe homeostasis in *Arabidopsis* is orchestrated by a multilayered transcriptional network centered on the bHLH transcription factor family (Gao et al., 2019), in which bHLH121/URI serves as the master integrator of Fe‐deficiency signals (Lei et al., 2020). Under Fe‐limiting conditions, bHLH121/URI activates a suite of key regulatory genes—including those encoding FIT, clade Ib bHLHs, PYE, IMA peptides, MYB10/72, and the BTS family—which collectively orchestrate Fe uptake and internal distribution (Gao, Robe, & Dubos, 2020; Hindt et al., 2017; Kim et al., 2019; Li et al., 2016; Palmer et al., 2013; Sivitz et al., 2011; Wang et al., 2013). Despite extensive characterization of this signaling cascade, additional downstream components essential for full acclimation to Fe deprivation remain to be identified. Our data position ANAC017 as a regulatory branch downstream of bHLH121/URI. Expression profiling revealed that Fe deficiency strongly induces *ANAC017* transcription in wild‐type plants, whereas loss of *ANAC017* function leads to hyperinduction of major Fe‐deficiency‐responsive genes, including *FIT*, *bHLH38/39*, *PYE*, and *IMA1* (Table 1). This enhanced transcriptional activation, together with the Fe‐sensitive phenotype of *anac017*, suggests that *ANAC017* operates within the canonical bHLH121/URI‐FIT signaling network rather than operating independently of it.

To define the positional relationship of ANAC017 within this network, we first performed yeast two‐hybrid (Y2H) assays to detect potential interactions between ANAC017 and the core components of the bHLH‐centered Fe regulatory module but failed to detect any physical interaction (Figure S4). Concurrently, yeast one‐hybrid (Y1H) screening preliminarily identified bHLH121/URI and MYB10 as potential upstream activators of *ANAC017* (Figure 7A). The direct transcriptional regulation of ANAC017 by bHLH121/URI was subsequently validated by both luciferase reporter assays and EMSAs (Figure 7C,D,F). By contrast, ANAC017 did not activate any tested Fe‐related regulators (Figure 7B), supporting its placement downstream rather than upstream within the network. Further analysis confirmed that Fe‐deficiency‐induced *ANAC017* expression is dependent on bHLH121 (Figure 7E). More importantly, overexpression of *ANAC017* substantially ameliorated the Fe‐sensitive phenotype of the *bhlh121/uri* mutant, alleviating chlorosis symptoms and increasing tissue Fe content (Figure 8). This genetic rescue provides compelling evidence that ANAC017 mediates an important branch of the bHLH121/URI‐dependent Fe‐deficiency response.

In addition, the preliminary Y1H screening showed that MYB10 may also regulate ANAC017, suggesting that ANAC017 may receive regulatory inputs from multiple components of the Fe‐deficiency signaling network. A similar regulatory pattern has been reported for MYB72, which is activated by both bHLH121/URI and FIT (Gao, Robe, Bettembourg, et al., 2020; Sivitz et al., 2012). Therefore, ANAC017 might integrate signals from different branches of the Fe‐deficiency network, which requires further experimental validation.

### ANAC017 may regulate Fe homeostasis via multiple pathways mediated by *MAT1*

To identify the downstream targets through which ANAC017 regulates Fe homeostasis, we analyzed the expression of genes involved in Fe uptake and transport in *anac017* mutants subjected to iron deficiency (Table 1). As expected, *FRO2*, *IRT1*, *FIT*, and *bHLH38/39* were strongly induced in Fe‐deprived wild‐type plants, which is consistent with a previous study (Lei et al., 2020). This induction was further enhanced in *anac017* mutants (Table 1), correlating with the more severe Fe‐sensitive phenotype observed in mutants (Figure 4). In addition, the transcriptional responses of several Fe transport‐related genes were markedly altered in the mutants. Fe deficiency suppressed *FRD3* and *YSL2* and induced *NAS1/2/4* in wild‐type roots, whereas these regulatory responses were abolished or attenuated in *anac017* mutants (Table 1), suggesting that ANAC017 may be involved in modulating the expression of a subset of Fe transport genes.

Based on these findings, we performed yeast one‐hybrid (Y1H) assays using known Fe‐responsive genes as candidate targets to identify direct targets of ANAC017. The results showed that ANAC017 can bind to the promoter of *MAT1*/*SAM1 in vitro* (Figure 9A). This finding was further supported by the effects of loss of *ANAC017* function on *MAT1/SAM1* transcript levels (Figure 9B) and by LUC reporter assays (Figure 9C,D), suggesting that *MAT1/SAM1* is a direct downstream target of ANAC017.

To determine whether MAT1/SAM1 is involved in the ANAC017‐dependent response to Fe deficiency, we first generated *MAT1/SAM1* overexpression transgenic lines and confirmed that they exhibited significantly enhanced tolerance to Fe‐deficiency stress (Figure S5). *MAT1/SAM1* overexpression effectively rescued the Fe‐deficiency‐sensitive phenotypes of the *anac017* mutant, as evidenced by alleviated root growth inhibition, reduced chlorosis symptoms, and improved tissue Fe accumulation (Figure 9B–H). Previous studies have confirmed that *MAT1/SAM1* encodes *S*‐adenosylmethionine synthetase, which catalyzes the production of *S*‐adenosylmethionine (SAM). SAM serves not only as the primary methyl donor for cellular methylation reactions but also as an essential precursor for the biosynthesis of ethylene (Vanderstraeten & Straeten, 2017) and NA (Curie et al., 2009). Notably, ethylene signaling has been shown to play an important role in the regulation of Fe homeostasis (Lingam et al., 2011), whereas NA functions as a key chelator for long‐distance Fe transport in the phloem (von Wirén et al., 1999). Based on the genetic evidence and previous findings, we propose that ANAC017 may mediate the synthesis and metabolism of SAM by regulating *MAT1*/*SAM1* expression, thereby contributing to plant adaptation to Fe deficiency through the roles of SAM as a precursor for ethylene and NA biosynthesis. It should be pointed out that whether ANAC017 indeed affects ethylene biosynthesis and NA‐mediated long‐distance Fe transport through *MAT1*/*SAM1* remains to be experimentally verified. Collectively, we propose a working model depicting how the ANAC017‐mediated pathway regulates the Fe‐deficiency response (Figure 10).

**Figure 10:**
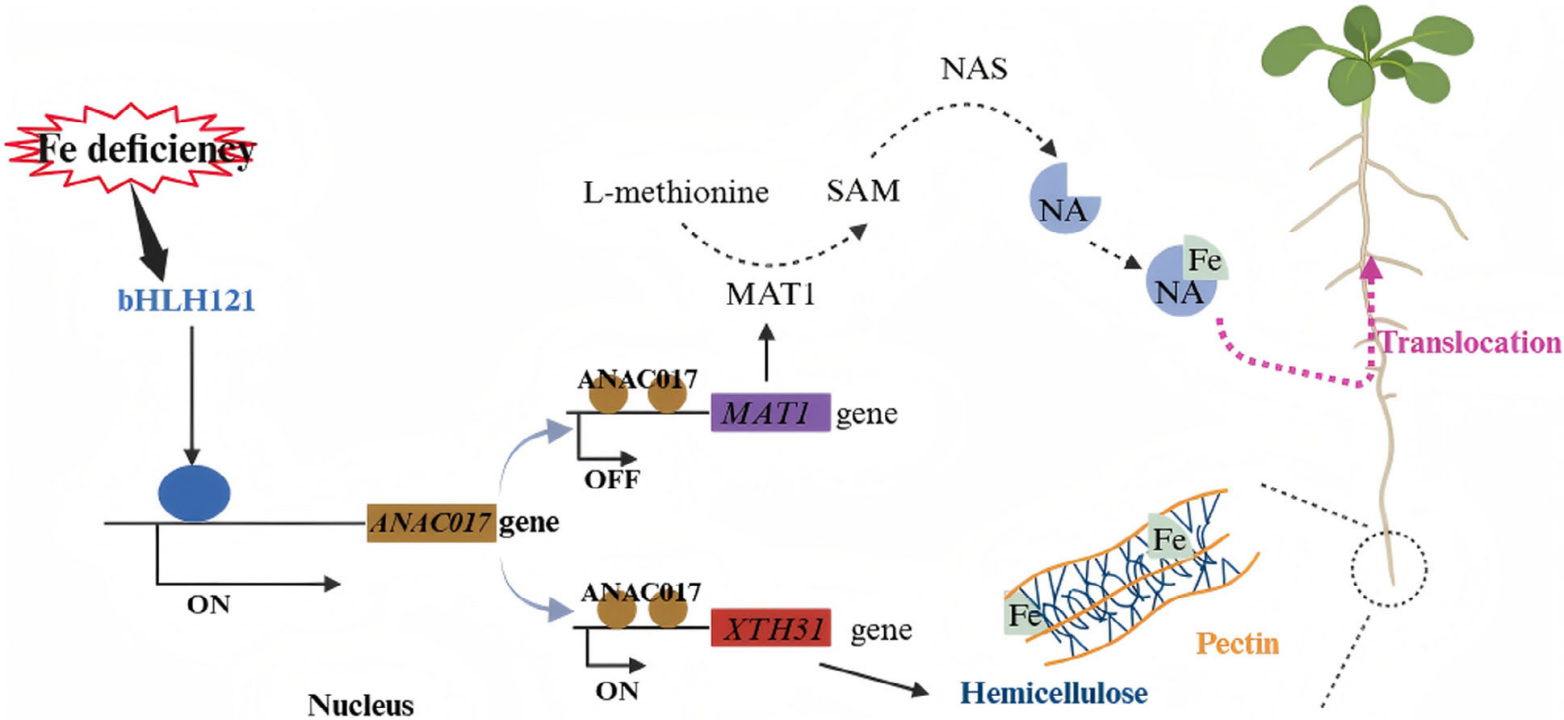
A proposed working model for the ANAC017 regulatory pathway under Fe deficiency. As a direct downstream target of bHLH121/URI, ANAC017 binds to the *MAT1/SMA1* promoter and regulates the expression of S‐adenosylmethionine synthetase (SAM), thereby modulating phloem Fe transport and ethylene signaling and contributing to Fe homeostasis in *Arabidopsis*. Furthermore, combined with previous findings that ANAC017 regulates cell wall heavy metal binding capacity through *XTH31*, suggesting that *ANAC017* may also contribute to the maintenance of Fe homeostasis by mediating the remobilization of cell wall‐bound Fe. Dashed lines represent hypothetical mechanisms that need further experimental validation.

## MATERIAL AND METHODS

### Vector construction and plant material generation

*Arabidopsis thaliana* (Columbia ecotype, Col‐0), the T‐DNA insertion mutants *anac017‐1* (SALK_044777) and *anac017‐2* (SALK_070231), the *ANAC017* overexpression lines (*ANAC017*‐OX‐1 and *ANAC017‐*OX‐2), and *ANAC017* complemented lines (*ANAC0*17‐COM1 and *ANAC*017‐COM2), and *ANAC017*‐GFP, *ProANAC017:GUS* seeds, and *MAT1*‐OX were obtained from our laboratory stock (Li et al., 2026; Tao et al., 2022). *bhlh21* (SALK_137976C) was obtained from the *Arabidopsis* Biological Resource Center (ABRC). The *ANAC017*‐OX/*bhlh121* and *MAT1*‐OX/*anac017* transgenic lines were generated via genetic crossing.

For the *ANAC017*‐GFP line, the full‐length coding sequence of *ANAC017* was cloned into the P‐super‐N‐GFP vector containing the cauliflower mosaic virus 35S promoter, and the constructed plasmids were transferred into *Arabidopsis* Col‐0 to obtain the *ANAC017*‐GFP lines (Tao et al., 2022). Constructs were introduced into *Agrobacterium tumefaciens* strain GV3101 using the freeze–thaw method as described previously (Zhu et al., 2012). *Arabidopsis* transformation was performed via the floral dip method. Transgenic seedlings were selected on Murashige and Skoog (MS) medium supplemented with hygromycin, and homozygous T3‐generation lines were used for all subsequent analyses.

### Plant culture conditions and experimental design

Wild‐type (Col‐0) and *anac017* mutant seedlings were grown hydroponically in standard nutrient solution for 14 days. Root samples were then harvested, rinsed thoroughly with deionized water to remove residual nutrients, weighed, and digested until clear. After digestion, the samples were diluted to a fixed volume and filtered, followed by quantification of B, Na, Mg, P, S, K, Ca, Mn, Fe, Cu, Zn, and Mo concentrations.

Surface‐sterilized seeds were sown uniformly on 1/2 MS medium supplemented with 1% (w/v) agar, plates were sealed with parafilm and placed vertically in a plant growth chamber under controlled conditions. When the primary roots reached approximately ≥1 cm, seedlings were transferred to 1/2 MS medium containing either 0 or 50 μM Fe (III)‐EDTA for 7 days. Subsequently, seedling phenotypes were photographed and primary root lengths were quantified. The growth chamber was maintained at 24°C with a 16 h light/8 h dark photoperiod.

For hydroponic experiments, seeds were placed on sterile centrifuge tubes fitted with sponge supports. After 4 weeks of growth in standard nutrient solution, seedlings showing uniform development were selected and subjected to normal or Fe‐deficient nutrient solutions. Hydroponic culture was maintained at 22°C with a 16 h light/8 h dark photoperiod.

Four‐week‐old Col‐0 plants, *ANAC017* overexpression lines, and *ProANAC017*:*GUS* transgenic seedlings were selected for time‐course analyses under Fe deficiency. Root tissues were harvested at 0, 0.5, 3, 6, 9, 12, and 24 h after the onset of Fe deprivation for RNA extraction, protein isolation, and GUS staining to monitor temporal changes in *ANAC017* transcript accumulation and promoter activity.

To assess physiological responses to Fe deficiency, 4‐week‐old seedlings of uniform size were transferred to either control or Fe‐deficient nutrient solution for 7 days. Subsequently, phenotypic documentation, SPAD chlorophyll index measurement, RNA extraction, FCR activity assay, and total Fe quantification were performed.

The 1/2 MS medium used for plate assays contained standard macronutrients and micronutrients at pH 4.5. Macronutrients were supplied as 6 mM KNO_3_, 4.0 mM Ca(NO_3_)_2_, 1 mM MgSO_4_, and 0.1 mM NH_4_H_2_PO_4_; micronutrients included 50 μM Fe(III)‐EDTA, 12.5 μM H_3_BO_3_, 1 μM MnSO_4_, 0.5 μM CuSO_4_, 1 μM ZnSO_4_, 0.1 μM H_2_MoO_4_, and 0.1 μM NiSO_4_.

For Cu, Zn, and Mn deficiency treatments, plate assays were performed on modified 1/2 MS medium. The control medium contained the complete 1/2 MS formulation as described above, whereas the deficiency media were prepared by selectively omitting CuSO_4_, ZnSO_4_, or MnSO_4_, respectively, from the basal medium, with all other components remaining unchanged. All media were solidified with 1% (w/v) agar, autoclaved, and dispensed into plates in a clean bench. Seeds were first germinated and grown on control medium. When primary roots reached approximately 1.2 cm in length, uniformly sized seedlings were selected and transferred to control or respective deficiency media. Seedlings were cultivated in a growth chamber for 5 days under a photoperiod of 16 h light/8 h dark at a light intensity of 120–150 μmol m^−2^ sec^−1^. Phenotypic changes were documented photographically, and root tissues were harvested for gene expression analysis. For each genotype, root tissues from five individual seedlings were pooled to constitute one biological replicate, and four independent biological replicates were collected per genotype under each treatment condition. Total RNA was extracted, and RT‐qPCR was performed to examine the transcript levels of target genes. The experiment was independently repeated three times with similar results.

### Measurement of SPAD value

Chlorophyll content in newly emerged leaves was estimated using a portable chlorophyll meter (SPAD‐502, Konica Minolta, Tokyo, Japan). For each plant, SPAD readings were collected from fully expanded young leaves, and mean values were calculated from multiple independent measurements to ensure accuracy.

### Determination of total Fe concentration

Root and shoots were harvested separately and rinsed three times with distilled water to remove surface contaminants. Samples were dried at 70°C for 2 days and weighed to determine dry biomass. For mineral analysis, dried tissues were digested in 2 ml concentrated HNO_3_ at 135°C using a digestion block until the solution became clear. The digests were then diluted to a final volume of 10 ml with distilled water and filtered through qualitative filter paper. Total Fe concentration was quantified using inductively coupled plasma‐atomic emission spectroscopy (ICP‐AES).

### Determination of FCR activity

The FCR activity was measured as described by Lei et al. (2014), with minor modifications. Roots were harvested after 7 days of growth under either control or Fe‐deficient conditions and immediately incubated in 5 ml of assay solution containing 0.5 mM CaSO_4_, 5 mM MES, 0.1 mM bathophenanthroline disulfonic acid (BPDS), and 100 μM Fe(III)‐ethylenediaminetetraacetate (Fe(III)‐EDTA). The pH of the solution was adjusted to 5.5 using NaOH. Samples were incubated for 1 h at room temperature on a shaker in the dark. After incubation, 200 μl of the reaction solution was collected, and the formation of the Fe^2+^‐BPDS_3_ complex was quantified by measuring absorbance at 535 nm using a microplate reader. A standard curve was generated using Fe^2+^ solutions (prepared from FeSO_4_) at defined concentrations by adding 0.5–2.5 μl of a 1 mg ml^−1^ Fe^2+^ stock to 5 ml of assay solution. FCR activity was calculated based on a standard curve, with assay solution lacking plant material used as the blank control.

### Fractionation of cell wall components and determination of cell wall‐associated Fe

Cell wall material was isolated following Zhu et al. (2016) with minor modifications. Root tissues were rinsed three times with deionized water and blotted dry. Samples were frozen in liquid nitrogen, ground to a fine powder, and transferred to 10 ml centrifuge tubes containing 8 ml of 75% (v/v) ethanol. The suspension was vortexed thoroughly and incubated for 20 min, followed by centrifugation at 5000 rpm for 10 min. The pellet was sequentially washed with acetone, methanol: chloroform (1:1, v/v), and methanol, with each wash involving vortexing and a 20‐min incubation prior to centrifugation. The resulting crude cell wall fraction was freeze‐dried and stored at 4°C until use.

Approximately 2 mg crude cell wall was suspended in 1 ml deionized water and incubated in a boiling water bath at 100°C for 1 h. After centrifugation at 12 000 rpm for 10 min, the supernatant was collected. This extraction step was repeated three times, and all supernatants were pooled. The remaining pellet was incubated with 1 ml of 24% KOH at room temperature for 12 h with gentle shaking. After centrifugation at 12 000 rpm for 10 min, the supernatant was collected, and the extraction was repeated once. The combined supernatants constituted the hemicellulose fraction (Zhu et al., 2012).

For quantification of cell wall‐bound Fe, approximately 2 mg of crude cell wall material was incubated with 1 ml of 2 M HCl for 3 days at room temperature with continuous shaking. Subsequently, the Fe concentration in the acid extracts was determined using ICP‐AES.

Fe levels in the extracted hemicellulose solutions were determined directly using ICP‐AES.

### Measurement of hemicellulose content

Hemicellulose content was estimated based on total soluble sugar content using the phenol‐sulfuric acid method (Dubois et al., 1956). Briefly, 200 μl of hemicellulose extract was mixed with 10 μl of phenol and 1 ml of concentrated sulfuric acid. The reaction mixture was incubated at room temperature for 15 min, followed by heating in a boiling water bath at 100°C for 15 min. Samples were rapidly cooled on ice, and absorbance was measured at 490 nm. Total sugar concentration was determined using a standard curve generated according to Dubois et al. (1956).

### RNA extraction and gene expression analysis

Root tissues from treated *Arabidopsis* seedlings were harvested and immediately flash‐frozen in liquid nitrogen. Frozen samples were ground to a fine powder, and total RNA was extracted using the Plant RNA extraction kit (TIANGEN, Beijing, China) according to the manufacturer's instructions. RNA quantity and purity were assessed using a Nano Drop spectrophotometer. About 1 μg of total RNA was used for first‐strand cDNA synthesis with the PrimeScript RT Reagent Kit (Takara, Shiga, Japan). The resulting cDNA was diluted and stored at −20°C for further investigation.

Quantitative qRT‐PCR was performed using SYBR Premix ExTaq (Takara Bio, Inc., Shiga, Japan). Initially, 10‐fold diluted cDNA served as a template in a 10 μl reaction containing 1 μl cDNA, 5 μl SYBR Premix ExTaq, 0.5 μl of forward and reverse primers, and 3 μl nuclease‐free water. Reaction was run with following thermal profile: 95°C for 1 min, followed by 40 cycles of 95°C for 15 sec, 55°C for 15 sec, and 72°C for 20 sec. Relative transcript abundance was calculated using the 2^−ΔΔCt^ method, with *tubulin* as the reference gene. Each experiment was conducted with 4 biological replicates and 3 technical replicates. Primers used in this study were listed in Table S1.

### Protein extraction and immunoblot analysis

For protein expression analysis during the Fe‐deficient time course: Frozen root tissues were ground in liquid nitrogen and homogenized in protein extraction buffer (Solarbio, China). Homogenates were incubated on ice for 20 min with intermittent vortexing and then centrifuged to obtain clarified protein extracts. Protein concentration was determined by the Bradford assay. Equal amounts of protein were mixed with 5 × SDS loading buffer and denatured at 100°C for 5 min. Samples were separated by SDS‐PAGE and transferred to membranes for immunoblotting. GFP‐specific antibody was used to detect ANAC017‐GFP fusion protein, and Actin‐specific antibody served as the internal reference.

### GUS staining

Four‐week‐old *ProANAC017:GUS* transgenic seedlings were transferred to Fe‐deficient 1/2 MS medium, and root samples were collected for staining at 0, 0.5, 3, 6, 9, 12, and 24 h after treatment onset. GUS activity was visualized using a histochemical staining kit (Solarbio, China). Samples were incubated at 37°C overnight and subsequently imaged using a Nikon AZ100 microscope.

### Yeast two‐hybrid assay

The full‐length coding sequence of *ANAC017* was amplified and cloned into the pGBKT7 vector to generate the bait construct (pGBKT7‐BD‐*ANAC017*). The full‐length coding sequences of *bHLH121/URI*, *bHLH101*, *bHLH38*, *bHLH39*, *bHLH115*, *MED25*, *bHLH105*, *bHLH34*, *bHLH100*, *bHLH104*, *FIT*, *PYE*, *IMA1*, *BSTL1*, and *MYB10* were inserted into the pGADT7 vector to generate prey constructs. Bait and prey plasmids were co‐transformed into the yeast strain AH109, and transformants were selected on SD‐Trp/‐Leu (‐T‐L) dropout medium for 3 days. Positive clones were then assayed on both ‐T‐L (control) and SD‐Trp/‐Leu/‐His/‐Ade+3‐AT (interaction selection) to test for protein–protein interactions. The primers used in this study are listed in Table S1.

### Yeast one‐hybrid assay

To investigate transcriptional regulatory relationships involving ANAC017, yeast one‐hybrid assays were performed with promoters of candidate genes (*bHLH121/URI*, *bHLH34*, *bHLH38*, *bHLH39*, *bHLH100*, *bHLH101*, *bHLH104*, *bHLH105*, *bHLH115*, *MYB10*, *FIT*, *PYE*, *BTSL1*, *IRT1*, *FRO2*, *FRD3*, *YSL2*, *NAS2*, *NAS4*, and *MAT1/SMA1*). Promoter fragments were cloned into the pLacZi reporter vector, and corresponding full‐length coding sequences were inserted into the pB42AD effector vector. Constructs were co‐transformed into the yeast strain EGY48, and positive transformants were selected on SD/–Ura/−Trp medium. Reporter activation was assessed on SD‐Trp/‐Ura + X‐gal medium containing chromogenic substrates (Tao et al., 2022). All primers used in this study are listed in Table S1.

### Dual‐luciferase reporter assay

The promoter of *ANAC017* or *MAT1* was fused to the *AtUbi*‐driven Renilla luciferase (rLUC) internal control vector, while the coding sequence of *bHLH121/URI* or *ANAC017* was cloned into the 190fLUC firefly luciferase reporter vector (primers listed in Table S1). Both constructs were co‐transformed into protoplasts. After 16 h of incubation, cells were harvested and lysed by adding 100 μl lysis buffer followed by vigorous shaking for 2 sec. The lysate was kept on ice for 5 min and then centrifuged at 1000 ***g*** for 2 min. A 20 μl aliquot of the supernatant was mixed with 100 μl luciferase substrate (Promega, Madison, WI, USA). Firefly and Renilla luciferase activities were measured using the PicaGene Dual‐LUC Assay Kit (PG‐DUAL SP; Toyo Ink, Osaka, Japan). Relative reporter activity was calculated by normalizing LUC readings to rLUC signals (Yoo et al., 2007).

### EMSA

To investigate the binding interaction between bHLH121 and the *ANAC01*7 promoter, *AtbHLH121* was directionally cloned into the pGEX‐4T‐1 expression vector carrying a GST tag. The recombinant plasmid was transformed into Escherichia coli BL21 (DE3) competent cells for heterologous protein expression. GST‐AtbHLH121 fusion protein expression was induced in LB medium with 1 mM isopropyl β‐D‐1‐thiogalactopyranoside (IPTG) at 28°C for 2 h. The harvested protein was affinity‐purified using glutathione agarose beads (GST tag). Oligonucleotide probes were labeled with biotin at the 3′ end using a commercial EMSA probe biotin labeling kit (Beyotime, Shanghai, China), and unlabeled (cold) oligonucleotides were used as competitors in the binding reaction. EMSA was then performed according to the manufacturer's instructions (Beyotime, EMSA kit). The purified recombinant protein was incubated with the biotin‐labeled probe. The EMSA binding reactions were performed in a total volume of 10 μl, containing 2 μl of 5 × EMSA/Gel‐shift binding buffer, 5 μl of purified AtbHLH121‐GST protein (omitted in the negative control), and 1 μl of biotin‐labeled probe. For the competition assays, the unlabeled specific competitor (Cold‐probe) and the mutated probe (Mutated‐probe) were added at the same concentration and equal volume (1 μl) as the biotin‐labeled probe (1:1 ratio). The reaction mixtures were adjusted to a final volume of 10 μl with nuclease‐free water. To eliminate potential non‐specific binding or allow the cold probe to react preferentially, the reaction mixture (excluding the biotin‐labeled probe) was thoroughly mixed and incubated at room temperature for 10 min prior to the addition of the biotin‐labeled probe. Subsequently, the biotin‐labeled probe was added, and the mixture was incubated at room temperature for an additional 20 min. After incubation, loading buffer was added to the prepared reaction mixtures. The samples were then loaded onto an EMSA gel and electrophoresed at 60 V for 100 min. Subsequently, the proteins and probes were electro‐transferred onto a membrane at 320 mA for 60 min. Following the transfer, the membrane was subjected to UV crosslinking for 1 min, and this crosslinking procedure was repeated 2–3 times. Finally, the biotin‐labeled probes were detected using a chemiluminescent method.

### Statistical analysis

All experiments were repeated independently at least twice, and the representative results are shown. Statistical differences between two groups were analyzed using Student's *t*‐test. For multiple comparisons, data were evaluated via one‐way or two‐way analysis of variance (ANOVA) followed by Tukey's post hoc test. Differences were considered statistically significant at *P* < 0.05 or *P* < 0.01. Significant differences are indicated by asterisks or different lowercase letters.

## AUTHOR CONTRIBUTIONS

XZ and FY designed the experiments; CT and QW performed the experiments, analyzed the data, and wrote the manuscript; XZ, FY, and RS revised the manuscript. All authors read and approved the manuscript.

## CONFLICT OF INTEREST

The authors declare no conflict of interest.

## Supporting information


**Table S1.** Primers used in the study.
**Figure S1.** Growth performance and expression of representative response genes in *anac017* mutants under Cu‐, Zn‐, and Mn‐deficient conditions.
**Figure S2.** *ANAC017* overexpression confers improved Fe‐deficiency tolerance.
**Figure S3.** The *ANAC017*‐complementing lines restores the *anac017* mutant phenotype to wild type levels.
**Figure S4.** Yeast two‐hybrid analysis of ANAC017.
**Figure S5.** The tolerance of *MAT1/SAM1* overexpression lines to Fe deficiency stress.

## Data Availability

The data supporting the study findings are available from the corresponding author upon reasonable request.
