# Supplementary material for: A bHLH121/URI–ANAC017–MAT1/SAM1 regulatory cascade controls iron homeostasis in Arabidopsis
Source: Plant J. 2026 Oct 14;128(1):e71157. doi: 10.1111/tpj.71157 (PMC13669638; doi:10.1111/tpj.71157)
Supplement: Supplementary file 1 [file TPJ-128-0-s001.docx]

**Table S1. Primers used in the study**

| **Description** | **Primer name** | **Primer sequence (5‘→3′)** |
| --- | --- | --- |
| *anac017-1* T-DNA | SALK_044777-LP | CTCTTTCCTCAGTGGCAACAG |
| *anac017-1* T-DNA | SALK_044777-RP | CAGGGAACTTCTGCAAATCTG |
| *anac017-2* T-DNA | SALK_070231-LP | GACTCATGCTTGTCTTGGAGC |
| *anac017-2* T-DNA | SALK_070231-RP | AACCAAATAACGGATCCGATC |
| *bhlh121* T-DNA | SALK_137976C-LP | TTCAAGCAGTGCTCTAGAGAGC |
| *bhlh121* T-DNA | SALK_137976C-RP | GAATTGATTTCCACGACGAAC |
| qRT-PCR | *AtACTIN*-F | GACCTTTAACTCTCCCGCTA |
| qRT-PCR | *AtACTIN*-R | GGAAGAGAGAAACCCTCGTA |
| qRT-PCR | *AtFRD3*-F | TTTTGTCGGGCGTTTAGG |
| qRT-PCR | *AtFRD3*-R | TTGCTGTGGCTGGTTGGT |
| qRT-PCR | *AtYSL2*-F | GGATACTTATTCTTCTCCCTTGTC |
| qRT-PCR | *AtYSL2*-R | CCATCGTTTTTTCCTGCC |
| qRT-PCR | *AtNAS1*-F | CATGATCTTCCACACAACGGAC |
| qRT-PCR | *AtNAS1*-R | CGACGTCATATTGGTCAAGGC |
| qRT-PCR | *AtIRT1*-F | GGTTGTATCCTCCAGGCTGAGT |
| qRT-PCR | *AtIRT1*-R | TGGTGTTGTTACCGCGAA AA |
| qRT-PCR | *AtFRO2*-F | GCCACATCTGCGTATCAAGTT |
| qRT-PCR | *AtFRO2*-R | TCCCAAACAAGCTACGACCA |
| qRT-PCR | *AtFIT*-F | CAGTCACAAGCGAAGAAACTCA |
| qRT-PCR | *AtFIT*-R | CTTGTAAAGAGATGGAGCAACACC |
| qRT-PCR | *AtANAC017-*F | TGAGGTTCAATGGAAAGGCT |
| qRT-PCR | *AtANAC017-*R | CCAGAAGATGGCACACAAAG |
| qRT-PCR | *AtbHLH121-*F | GAAAGCAGAGGACTCCAACGA |
| qRT-PCR | *AtbHLH121-*R | GTCTTCTCTGGCCTTTGCAATG |
| qRT-PCR | *AtbHLH38-*F | ACGGTGCCGGAGATAACCTA |
| qRT-PCR | *AtbHLH38-*R | GTCGGTCACGTTCACTAGCA |
| qRT-PCR | *AtbHLH39-*F | CCGTTCATGTCTTCCTGCCT |
| qRT-PCR | *AtbHLH39-*R | GCCTTTGGTGGCTGCTTAAC |
| qRT-PCR | *AtPYE-*F | CAGGACTTCCCATTTTCCAAG |
| qRT-PCR | *AtPYE-*R | CTTGTGTCTGGGGATCAGGTT |
| qRT-PCR | *AtMYB10-*F | GGGGAAATCTTGGTGGAGCA |
| qRT-PCR | *AtMYB10-*R | AGGAGGAACCTGGCTATCGT |
| qRT-PCR | *AtNAS2*-F | CGGTCCGATGCCACTTACTT |
| qRT-PCR | *AtNAS2*-R | TTTGAAGCGAGTGTGTTGGC |
| qRT-PCR | *AtNAS4*-F | CTTCCGTCGTTCTTGCCTCT |
| qRT-PCR | *AtNAS4*-R | TTCGCTGATGGGTCGATGTC |
| qRT-PCR | *AtIMA1*-F | GTGGCTTTCTCTTTACACCTCTTCATTGC |
| qRT-PCR | *AtIMA1*-R | CGCAGCAGGAGCATAATCATAGCC |
| qRT-PCR | *AtMAT1*-F | TAGGGCTCTTGTTCAGGTCT |
| qRT-PCR | *AtMAT1*-R | AGTGTCCGTAAGCCGCAGTT |
| qRT-PCR | *AtXTH31*-F | TAGTGTTGTGCCCTAGTAGTGG |
| qRT-PCR | *AtXTH31*-R | AGAGCCCCATAAAGTACGGAAT |
| qRT-PCR | *AtYSL1-F* | TGGCTTCCACACACAAGGAG |
| qRT-PCR | *AtYSL1-R* | ACCACTGGAAGAAACCCCAC |
| qRT-PCR | *AtOPT3-F* | AAGCTTACTATAAACAGAGCCTTAGCTT |
| qRT-PCR | *AtOPT3-R* | ACAGGATCAACAAGGTACCTCCTC |
| qRT-PCR | *AtCOPT1-F* | TCCACCGGAGATTCGGCTAA |
| qRT-PCR | *AtCOPT1-R* | GCACAACCTGAGGGAGGAAC |
| qRT-PCR | *AtCOPT2-F* | CCTTTCGTATTTGGTGATGCT |
| qRT-PCR | *AtCOPT2-R* | AAACACCTGCGTTAAAGGAC |
| qRT-PCR | *AtCOPT3-F* | GGTCTGTCTCGCCGTCATTT |
| qRT-PCR | *AtCOPT3-R* | ATCCAAAACCAGCCATCGCA |
| qRT-PCR | *AtCOPT4-F* | TGGCGTTCTTAGCTGAGTGG |
| qRT-PCR | *AtCOPT4-R* | CGGAAAACAGCGAACCCAAG |
| qRT-PCR | *AtCOPT5-F* | ATCAATACCTCGAGAATCGCCGCA |
| qRT-PCR | *AtCOPT5-R* | AGCTGCAAGCATCAGCAAGTAACC |
| qRT-PCR | *AtCOPT6-F* | AGGGGCTCGTTCAGACCGCA |
| qRT-PCR | *AtCOPT6-R* | CCACGGCGAATCCGGCGATA |
| qRT-PCR | *AtNRAMP1-F* | GTAGCCACTTCTCTTCTTATTTCAAG |
| qRT-PCR | *AtNRAMP1-R* | ACCTTTATTCTAGTACACTATCGAAAG |
| qRT-PCR | *AtNRAMP2-F* | GCGGAAGCTCTGGTTATTCA |
| qRT-PCR | *AtNRAMP2-R* | AACAACCCCATAGCTGTTGC |
| qRT-PCR | *AtNRAMP3-F* | CCGAGTCCAAGAAGCGCTAA |
| qRT-PCR | *AtNRAMP3-R* | CCGCGTTAACCAAACCGATG |
| qRT-PCR | *AtNRAMP4-F* | AACAATGATCGTGGCGCTTG |
| qRT-PCR | *AtNRAMP4-R* | AGTGGGATCACAGCGAAAGG |
| qRT-PCR | *AtNRAMP6-F* | GAGTCCTCGTCCACACATTTCC |
| qRT-PCR | *AtNRAMP6-R* | TTTAAGTCTCCTATAACCGCTACTC |
| qRT-PCR | *AtZIP1-F* | AGCATAGACACCATAAAGCCACTC |
| qRT-PCR | *AtZIP1-R* | CCAACCCTATTCCTATCCCAAGT |
| qRT-PCR | *AtZIP2-F* | CGTCGTGAGATAAAGAGTGGTGTT |
| qRT-PCR | *AtZIP2-R* | TGTCCATAGGTTTCTCCAAGCG |
| qRT-PCR | *AtZIP3-F* | GCCTTAATGTTTCATCAATGCTTC |
| qRT-PCR | *AtZIP3-R* | GCCTTAATGTTTCATCAATGCTTC |
| qRT-PCR | *AtHMA2-F* | AACTGAAATCGTTGGGAATTAAGA |
| qRT-PCR | *AtHMA2-R* | TGGCCCTTCTTCTCTCTTTAACTG |
| qRT-PCR | *AtHMA4-F* | CCGCAATGCTAACGGGAGATA |
| qRT-PCR | *AtHMA4-R* | GCGGTTGGTCCCTCTTTCTTA |
| Yeast one hybrid assay | *AtANAC017-*LACZ*-*F | ATCTGTCGACCTCGAGTTTTCTAACACGGTGAATGTAACAGGTC |
| Yeast one hybrid assay | *AtANAC017-*LACZ*-*R | GAGCACATGCCTCGAGCTACGTAACAAATCAAAACCGATCCCTC |
| Yeast one hybrid assay | *AtbHLH121-*PB42AD*-*F | TGCCTCTCCCGAATTCATGGGGATAAGAGAAAATGGAATAA |
| Yeast one hybrid assay | *AtbHLH121-*PB42AD*-*R | CGAGTCGGCCGAATTCTCATTTTGCATCATCAGGTTTTTGG |
| Yeast one hybrid assay | *AtbHLH34-*PB42AD*-*F | TGCCTCTCCCGAATTCATGTATCCATCAATCGAAGACG |
| Yeast one hybrid assay | *AtbHLH34-*PB42AD*-*R | CGAGTCGGCCGAATTCTTAAGCAACAGGAGGAAGATTTTT |
| Yeast one hybrid assay | *AtbHLH104-*PB42AD*-*F | TGCCTCTCCCGAATTCATGTATCCTTCTCTCGACGATGATT |
| Yeast one hybrid assay | *AtbHLH104-*PB42AD*-*R | CGAGTCGGCCGAATTCTTAAGCAGCAGGAGGCCTGAGTTC |
| Yeast one hybrid assay | *AtbHLH105-*PB42AD*-*F | TGCCTCTCCCGAATTC ATGGTGTCACCCGAAAACGCTAATT |
| Yeast one hybrid assay | *AtbHLH105-*PB42AD*-*R | CGAGTCGGCCGAATTC TTAAGCAACAGGAGGACGAAGGACA |
| Yeast one hybrid assay | *AtbHLH115-*PB42AD*-*F | TGCCTCTCCCGAATTC ATGGTGTCTCCGGAGAATACGAACT |
| Yeast one hybrid assay | *AtbHLH115-*PB42AD*-*R | CGAGTCGGCCGAATTC TTAAGCAACTGGAGGACGAAGGACA |
| Yeast one hybrid assay | *AtbHLH100-*PB42AD*-*F | TGCCTCTCCCGAATTCATGTGTGCACTTGTCCCTCCATTAT |
| Yeast one hybrid assay | *AtbHLH100-*PB42AD*-*R | CGAGTCGGCCGAATTCTCATGTAAACGAGTGTCCACATTTC |
| Yeast one hybrid assay | *AtbHLH101-*PB42AD*-*F | TGCCTCTCCCGAATTCATGTGTACCTTAACGCCAATGT |
| Yeast one hybrid assay | *AtbHLH101-*PB42AD*-*R | CGAGTCGGCCGAATTCTTATGATTGGCGTAATCCCAAGAGC |
| Yeast one hybrid assay | *AtbHLH38-*PB42AD*-*F | TGCCTCTCCCGAATTCATGTGTGCATTAGTCCCTTCA |
| Yeast one hybrid assay | *AtbHLH38-*PB42AD*-*R | CGAGTCGGCCGAATTCCTAGTTAAACGAGTTTTCACATT |
| Yeast one hybrid assay | *AtbHLH39-*PB42AD*-*F | TGCCTCTCCCGAATTCATGTGTGCATTAGTACCTCCATTGT |
| Yeast one hybrid assay | *AtbHLH39-*PB42AD*-*R | CGAGTCGGCCGAATTCTCATATATATGAGTTTCCACATTCC |
| Yeast one hybrid assay | *AtMYB10-*PB42AD*-*F | TGCCTCTCCCGAATTCATGGGGAATAGAAGAGCACCATGCT |
| Yeast one hybrid assay | *AtMYB10-*PB42AD*-*R | CGAGTCGGCCGAATTCCTAGCAAGTTTGGTTATCCAAGAAA |
| Yeast one hybrid assay | *AtMYB72-*PB42AD*-*F | TGCCTCTCCCGAATTCATGGGGAAAGGAAGAGCACCATGC |
| Yeast one hybrid assay | *AtMYB72-*PB42AD*-*R | CGAGTCGGCCGAATTCTCATAGACATACTTCTCCGACGAAA |
| Yeast one hybrid assay | *AtFIT-*PB42AD*-*F | TGCCTCTCCCGAATTCATGGAAGGAAGAGTCAACGCTCTGT |
| Yeast one hybrid assay | *AtFIT-*PB42AD*-*R | CGAGTCGGCCGAATTCTCAAGTAAATGACTTGATGAATTCA |
| Yeast one hybrid assay | *AtPYE-*PB42AD*-*F | TGCCTCTCCCGAATTCATGGTATCGAAAACTCCTTCTACAT |
| Yeast one hybrid assay | *AtPYE-*PB42AD*-*R | CGAGTCGGCCGAATTCTCATTCACTGGCTTTCAGCCGCTCT |
| Yeast one hybrid assay | *AtANAC017-*PB42AD*-*F | TGCCTCTCCCGAATTCATGGCGGATTCTTCACCCGATTCGT |
| Yeast one hybrid assay | *AtANAC017-*PB42AD*-*R | CGAGTCGGCCGAATTCCTAGTCTTTCAAGAGAAGACTTCTACC |
| Yeast one hybrid assay | *AtYSL2-*LACZ*-*F | ATCTGTCGACCTCGAGCATAGAAACAAGACAAACCACAAAA |
| Yeast one hybrid assay | *AtYSL2-*LACZ*-*R | GAGCACATGCCTCGAGAAATCGTTAGGTTGCTTCTTGTACA |
| Yeast one hybrid assay | *AtNAS2-*LACZ*-*F | ATCTGTCGACCTCGAGTTATTCACACTACCTTGAGCAGAGA |
| Yeast one hybrid assay | *AtNAS2-*LACZ*-*R | GAGCACATGCCTCGAGACTTGGAAAAAAGCTTGTGTTGTTG |
| Yeast one hybrid assay | *AtNAS4-*LACZ*-*F | ATCTGTCGACCTCGAGAGTTTTGTTTTCTACACTTTACCAG |
| Yeast one hybrid assay | *AtNAS4-*LACZ*-*R | GAGCACATGCCTCGAGTGAAGTGTTAATTTTTAAATTTC |
| Yeast one hybrid assay | *AtFRD3-*LACZ*-*F | ATCTGTCGACCTCGAGCACACGCCTATAAATAATCCCCTCT |
| Yeast one hybrid assay | *AtFRD3-*LACZ*-*R | GAGCACATGCCTCGAGGAGGGAAAATAGGTACGCATAAACA |
| Yeast one hybrid assay | *AtMYB72-*LACZ*-*F | ATCTGTCGACCTCGAGGCACTTCCTTATTCTGTGATTTGAG |
| Yeast one hybrid assay | *AtMYB72-*LACZ*-*R | GAGCACATGCCTCGAGTCTTATTACACTACTTTCTTCTCTA |
| Yeast one hybrid assay | *AtFRO2-*LACZ*-*F | ATCTGTCGACCTCGAGGGGGATACACAGTCCAGATCATACG |
| Yeast one hybrid assay | *AtFRO2-*LACZ*-*R | GAGCACATGCCTCGAGCTATCTCTCTCTCTCTCGCTCGCTA |
| Yeast one hybrid assay | *AtMAT1-*LACZ*-*F | ATCTGTCGACCTCGAGTCTCTCGCTTCTTCTTATTCTTCAA |
| Yeast one hybrid assay | *AtMAT1-*LACZ*-*R | GAGCACATGCCTCGAGTAAACAGAATCAAAGTGTCCCAATC |
| Yeast one hybrid assay | *AtbHLH100-*LACZ*-*F | ATCTGTCGACCTCGAGGGATTCATAAATGGGTCTGCT |
| Yeast one hybrid assay | *AtbHLH100-*LACZ*-*R | GAGCACATGCCTCGAGCACGGACACAAGAAAAATACT |
| Yeast one hybrid assay | *AtbHLH101-*LACZ*-*F | ATCTGTCGACCTCGAGTTAGGGTTTTTTCTTCTTTTA |
| Yeast one hybrid assay | *AtbHLH101*-LACZ*-*R | GAGCACATGCCTCGAGATGGAATCTATATATCTATGT |
| Yeast one hybrid assay | *AtbHLH38-*LACZ*-*F | ATCTGTCGACCTCGAGAACGGCGGGTTTCTTGATTTT |
| Yeast one hybrid assay | *AtbHLH38-*LACZ*-*R | GAGCACATGCCTCGAGAGTGTTTTTATTCGTTGAGAT |
| Yeast one hybrid assay | *AtbHLH39-*LACZ*-*F | ATCTGTCGACCTCGAGATTGTTCTCAGGAAGAGTTGG |
| Yeast one hybrid assay | *AtbHLH39-*LACZ*-*R | GAGCACATGCCTCGAGTATAAGTGTTATACGACTTGT |
| Yeast one hybrid assay | *AtFIT-*LACZ*-*F | ATCTGTCGACCTCGAGTTTTGATGTGATGTGAGATGAGAAG |
| Yeast one hybrid assay | *AtFIT-*LACZ*-*R | GAGCACATGCCTCGAGAGTTTATATAATAGAGTGAGT |
| Yeast one hybrid assay | *AtbHLH115-*LACZ*-*F | ATCTGTCGACCTCGAGCAACTTCTCTCTCTTGCTCTTCACC |
| Yeast one hybrid assay | *AtbHLH115-*LACZ*-*R | GAGCACATGCCTCGAGTCTCTCTCAATCGGTTTCTATT |
| Yeast one hybrid assay | *AtBTSL1-*LACZ*-*F | ATCTGTCGACCTCGAGATCTGAGGCATAACCAGCTCCAAAT |
| Yeast one hybrid assay | *AtBTSL1-*LACZ*-*R | GAGCACATGCCTCGAGATTTTCGTAGTCGTCACTCTTCTGC |
| Yeast two hybrid assay | *AtbHLH121-*AD*-*F | ATGGAGGCCAGTGAATTCATGGGGATAAGAGAAAATGGAATAA |
| Yeast two hybrid assay | *AtbHLH121-*AD*-*R | AGCTCGAGCTCGATGGATCCTCATTTTGCATCATCAGGTTTTTGG |
| Yeast two hybrid assay | *AtbHLH34-*AD*-*F | ATGGAGGCCAGTGAATTCATGTATCCATCAATCGAAGACG |
| Yeast two hybrid assay | *AtbHLH34-*AD*-*R | AGCTCGAGCTCGATGGATCCTTAAGCAACAGGAGGAAGATTTTT |
| Yeast two hybrid assay | *AtbHLH100-*AD*-*F | ATGGAGGCCAGTGAATTCATGTGTGCACTTGTCCCTCCATTAT |
| Yeast two hybrid assay | *AtbHLH100-*AD*-*R | AGCTCGAGCTCGATGGATCCTCATGTAAACGAGTGTCCACATTTC |
| Yeast two hybrid assay | *AtbHLH101-*AD*-*F | ATGGAGGCCAGTGAATTCATGTGTACCTTAACGCCAATGT |
| Yeast two hybrid assay | *AtbHLH101-*AD*-*R | AGCTCGAGCTCGATGGATCCTTATGATTGGCGTAATCCCAAGAGC |
| Yeast two hybrid assay | *AtbHLH115-*AD*-*F | ATGGAGGCCAGTGAATTCATGGTGTCTCCGGAGAATACGAACT |
| Yeast two hybrid assay | *AtbHLH115-*AD*-*R | AGCTCGAGCTCGATGGATCCTTAAGCAACTGGAGGACGAAGGACA |
| Yeast two hybrid assay | *AtbHLH104-*AD*-*F | ATGGAGGCCAGTGAATTCATGTATCCTTCTCTCGACGATGATT |
| Yeast two hybrid assay | *AtbHLH104-*AD*-*R | AGCTCGAGCTCGATGGATCCTTAAGCAGCAGGAGGCCTGAGTTC |
| Yeast two hybrid assay | *AtbHLH105-*AD*-*F | ATGGAGGCCAGTGAATTCATGGTGTCACCCGAAAACGCTAATT |
| Yeast two hybrid assay | *AtbHLH105-*AD*-*R | AGCTCGAGCTCGATGGATCCTTAAGCAACAGGAGGACGAAGGACA |
| Yeast two hybrid assay | *AtPYE-*AD*-*F | ATGGAGGCCAGTGAATTCATGGTATCGAAAACTCCTTCTACAT |
| Yeast two hybrid assay | *AtPYE-*AD*-*R | AGCTCGAGCTCGATGGATCCTCATTCACTGGCTTTCAGCCGCTCT |
| Yeast two hybrid assay | *AtMYB10-*AD*-*F | ATGGAGGCCAGTGAATTCATGGGGAATAGAAGAGCACCATGCT |
| Yeast two hybrid assay | *AtMYB10-*AD*-*R | AGCTCGAGCTCGATGGATCCCTAGCAAGTTTGGTTATCCAAGAAA |
| Yeast two hybrid assay | *AtWRKY46-*AD*-*F | ATGGAGGCCAGTGAATTCATGATGATGGAAGAGAAACTTGTGA |
| Yeast two hybrid assay | *AtWRKY46-*AD*-*R | AGCTCGAGCTCGATGGATCCCTACGACCACAACCAATCCTGTCCG |
| Yeast two hybrid assay | *AtMED25-*AD*-*F | ATGGAGGCCAGTGAATTCATGCAAGGTCCACGAAGCACTGGTG |
| Yeast two hybrid assay | *AtMED25-*AD*-*R | AGCTCGAGCTCGATGGATCCTTATGTCGATAAAGCCACAGTCTTA |
| Yeast two hybrid assay | *AtBTSL1-*AD*-*F | ATGGAGGCCAGTGAATTCATGGGAGGCGGAAATCTTCATTC |
| Yeast two hybrid assay | *AtBTSL1-*AD*-*R | AGCTCGAGCTCGATGGATCCCTAAAGGAGCCTTGAGTTGTAGGAG |
| Yeast two hybrid assay | *AtIMA1-*AD*-*F | ATGGAGGCCAGTGAATTCACCAAACTCACATCAACATTTGAAG |
| Yeast two hybrid assay | *AtIMA1-*AD*-*R | AGCTCGAGCTCGATGGATCCCCAATTCAATAATGTAGTACTAGTC |
| Yeast two hybrid assay | *AtbHLH38-*AD*-*F | ATGGAGGCCAGTGAATTCATGTGTGCATTAGTCCCTTCA |
| Yeast two hybrid assay | *AtbHLH38-*AD*-*R | AGCTCGAGCTCGATGGATCCCTAGTTAAACGAGTTTTCACATT |
| Yeast two hybrid assay | *AtbHLH39-*AD*-*F | ATGGAGGCCAGTGAATTCATGTGTGCATTAGTACCTCCA |
| Yeast two hybrid assay | *AtbHLH39-*AD*-*R | AGCTCGAGCTCGATGGATCCTCATATATATGAGTTTCCACATTCC |
| Yeast two hybrid assay | *AtFIT-*AD*-*F | ATGGAGGCCAGTGAATTCATGGAAGGAAGAGTCAACGCTCTGT |
| Yeast two hybrid assay | *AtFIT-*AD*-*R | AGCTCGAGCTCGATGGATCCTCAAGTAAATGACTTGATGAATTCA |
| Yeast two hybrid assay | *AtANAC017-*BD*-*F | TCAGAGGAGGACCTGCATAATGGCGGATTCTTCACCCGATTCGT |
| Yeast two hybrid assay | *AtANAC017-*BD*-*R | ATGCGGCCGCTGCAGGTCGACTAGTCTTTCAAGAGAAGACTTCTACC |
| EMSA assay | *AtANAC017*-probe-F | TAAGCAGAATTTTTGAACCTCTGTCGTGTCAGACAAGAATGTTGGAAAAA |
| EMSA assay | *AtANAC017*-probe-F | TTTTTCCAACATTCTTGTCTGACACGACAGAGGTTCAAAAATTCTGCTTA |
| EMSA assay | *AtANAC017*-mutated-probe-F | TAAGCAGAATTTTTGAACCTCTAAAAAATCAGACAAGAATGTTGGAAAAA |
| EMSA assay | *AtANAC017*-mutated-probe-F | TTTTTCCAACATTCTTGTCTGATTTTTTAGAGGTTCAAAAATTCTGCTTA |
| EMSA assay | *AtbHLH121*-GST-F | GCGTGGATCCCCGGAATTCATGGGGATAAGAGAAAATGGAATAA |
| EMSA assay | *AtbHLH121*-GST-R | TGCGGCCGCTCGAGTCGACTTTTGCATCATCAGGTTTTTGGCCA |

**Figure S1.** Growth performance and expression of representative response genes in *anac017* mutants under Cu-, Zn-, and Mn-deficient conditions.

**(A)** Phenotypes of Col-0 and *anac017* under control, Cu-, Mn-, and Zn-deficient conditions. Seedlings grown on control medium (1/2 MS medium) until root length reached 1.2 cm were selected and transferred to Cu-, Mn-, Zn-deficient and control media for 5 days, after which phenotypic changes were observed. Scale bar=1cm.

**(B-G)** Expression levels of Cu-responsive genes in roots of Col-0 and *anac017* before and after copper deficiency treatment: *COPT1* (B), *COPT2* (C), *COPT3* (D), *COPT4* (E), *COPT5* (F), and *COPT6* (G).

**(H-L)** Expression levels of Mn-responsive genes in roots of Col-0 and *anac017* before and after copper deficiency treatment: *NRAMP1* (H), *NRAMP2* (I), *NRAMP3* (J), *NRAMP4* (K) and *NRAMP6* (L).

**(M-Q)** Expression levels of Zn-responsive genes in roots of Col-0 and *anac017* before and after copper deficiency treatment: *ZIP1* (M), *ZIP2* (N), *ZIP3* (O), *HMA2* (P) and *HMA4* (Q).

For each genotype, root tissues from five individual seedlings were pooled to constitute one biological replicate, and five independent biological replicates were collected per genotype under each treatment condition. RNA was extracted, and the transcript levels of the target genes were determined by RT-qPCR. The experiment was independently repeated three times with similar results. Different letters above the bars indicate significant differences, as determined by two-way ANOVA followed by Tukey's post hoc test (*P*<0.05).

**Figure S2.** *ANAC017* overexpression confers improved Fe-deficiency tolerance.
**(A-B)** Phenotype (A) and primary root length (B) of wild-type (Col-0) and *ANAC017*-OX lines under control and Fe-deficient conditions in plate assays. Data are means±SD (n=10 individual plants). Scale bar=1cm. Seedlings grown on control medium until root length reached 1.2 cm were selected and transferred to Fe-deficient and control media for 7 days, after which phenotypic changes were observed.

**(C-D)** Phenotype (C) and SPAD values (D) of Col-0 and *ANAC017*-OX lines under control and Fe-deficient conditions in hydroponic experiments. Data are means±SD (n=8 individual plants). Scale bar= 2cm.

**(E-G)** Root Fe concentration (E), shoot Fe concentration (F), and root available Fe content (G) of Col-0 and *anac017* under control and Fe-deficient conditions. Uniformly grown 4-week-old Arabidopsis seedlings were transferred to and treated in either +Fe or -Fe nutrient solution for 7 days. Values are expressed as mean ± SD (n=4 individual plants), and the experiment was repeated at least three times independently. Different letters indicate significant differences, as determined by two-way ANOVA followed by Tukey's post hoc test (*P*<0.05).

**Figure S3.** The *ANAC017*-complementing lines restores the *anac017* mutant phenotype to wild type levels.
**(A)** Phenotypes of Col-0, the *anac017* mutant, and *ANAC17*-complementing lines under control and Fe-deficient treatment, Scale bar=2 cm.

**(B)** SPAD values of the respective lines after treatment.

Uniform 4-week-old seedlings were treated in control (+Fe) or Fe-deficient (-Fe) medium for 7 days. Data represent mean±SD (n=8 individual plants). Different letters above the bars indicate significant differences, as determined by two-way ANOVA followed by Tukey's post hoc test (*P<*0.05).

**Figure S4.** Yeast two-hybrid analysis of ANAC017.

A yeast two-hybrid assay was performed by co-transforming the bait construct (pGBKT7-BD-*ANAC017*) with prey vectors (pGADT7-AD-*bHLH121/URI* / *bHLH34* / *bHLH104* / *bHLH105* / *bHLH115* / *bHLH38* / *bHLH39* / *bHLH100* / *bHLH101* / *FIT* / *PYE* / *IMA1* / *MYB10* / *MED25* / *BSTL1*) into yeast strain AH109. Protein-protein interactions were evaluated by comparing yeast growth on SD/-Trp/-Leu and SD/-Trp/-Leu/-His/-Ade +3-AT selection media. No protein-protein interactions were observed under the experimental conditions.

**Figure S5**. The tolerance of *MAT1/SAM1* overexpression lines to Fe deficiency stress.

**(A)** The expression level of *MAT1/SAM1* in *MAT1*-OX lines. The selected four stable genetic *MAT1*-OX lines were cultured in normal nutrient environment for 15 days. Four seedlings were randomly selected from each line to extract RNA from root tissue, and the transcription level of *MAT1/SAM1* was analyzed by qPCR. Significant differences were determined by one-way ANOVA followed by Tukey's post hoc test (*P<*0.05).

**(B-C)** growth phenotype (B) and main root (C) length of *MAT1*-OX lines under normal and Fe deficiency conditions. When the primary roots had grown to ≥1.2 cm, the seedlings were transferred to the corresponding media and grown for 7 days. Data are means±SD (n=10 individual plants). Significant differences were determined by two-way ANOVA followed by Tukey's post hoc test (*P<*0.05).
